# Artifact-reference multivariate backward regression (ARMBR): a novel method for EEG blink artifact removal with minimal data requirements

**DOI:** 10.1088/1741-2552/ade566

**Published:** 2025-06-26

**Authors:** L Alkhoury, G Scanavini, S Louviot, A Radanovic, S A Shah, N J Hill

**Affiliations:** 1 Department of Radiology, Weill Cornell Medicine, New York, NY 10065, United States of America; 2 National Center for Adaptive Neurotechnologies, Stratton VA Medical Center, Albany, NY, United States of America; 3 Electrical and Computer Engineering Department, State University of New York at Albany, Albany, NY, United States of America

**Keywords:** artifact-removal, automatic blink detection, electroencephalogram (EEG), electrooculogram (EOG), online and offline processing

## Abstract

*Objective*. We present a novel and lightweight method that removes ocular artifacts from electroencephalography (EEG) recordings while demanding minimal training data. *Approach*. A robust, cross-validated thresholding procedure automatically detects the times at which eye blinks occur, then a linear scalp projection is estimated by regressing a simplified time-locked reference signal against the multi-channel EEG. *Main results*. Performance was compared against four commonly-used and readily available blink removal methods: signal subspace projection and forward regression (Reg) from the MNE toolbox, EEGLab’s independent component analysis (ICA) combined with ICLabel for automated component identification, and Artifact Subspace Reconstruction (ASR) Python implementation compatible with MNE. On semi-synthetic blink-contaminated EEG data, our method exhibited better reconstruction of the ground truth than the two MNE native methods, and comparable (or better in some scenarios) performance to ASR algorithm and ICA+IClabel. We also examined a real EEG dataset from 16 human participants, where the ground truth was unknown. Our method affected contaminated channels in blink intervals more than the two MNE native methods and ASR, while having a smaller impact on non-blink intervals, uncontaminated channels, and higher-frequency amplitudes, than the two MNE methods; its performance was again similar to ICA+ICLabel. On a second real dataset from 42 human participants, we showed that ARMBR removed the unwanted blink artifacts while successfully preserving the desired event-related-potential signals. *Significance*. The proposed algorithm exhibited comparable, and in some scenarios better performance relative to readily-available implementations of other widely-used methods. Another feature of our method is its potential as method for online applications. Therefore, it stands to make valuable contributions towards the automation of neural-engineering technologies and their translation from laboratory to clinical and other real-world usage.

## 1. Introduction

Electroencephalography (EEG) non-invasively captures cerebral electrical activity, an important feature that lends it as the most common technique in clinical and non-clinical settings [42]. EEG is employed in a broad range of applications including the study of motor and cognitive function, assessing cognitive workload, attention levels and other neural dynamics [33], sleep patterns [41], brain–computer interfaces (BCIs) [47], neurofeedback [20], and clinical characterization and diagnosis of various cerebral disorders (primarily epilepsy and stroke) [1, 15], to name just a few. The waveforms recorded during an EEG session comprise both neural and non-neural signals. The non-neural sources encompass induced electrical signals originating from the recording environment (e.g. line noise), along with biological signals (e.g. blinks, eye movements, muscle activity, and skin potentials) and are commonly considered artifacts. Artifacts present multiple challenges in neuroscience experiments, including reducing the signal-to-noise ratio (SNR) of targeted waveforms. The typical dominant fraction consists of ocular artifacts, arising from blinks and eye movements, both of which induce modifications in sensory input. Artifact handling is typically divided into two phases, namely, (a) detection, followed by either (b) rejection or correction.

Considering that the rejection of EEG data based on the presence of artifacts may lead to substantial information loss, numerous methods and algorithms have been developed over the years for artifact correction; however, optimal correction procedures remain undetermined due to the wide variety of EEG data applications. The method proposed in this paper is tailored for the subspace that includes real-time applications in both clinical and non-clinical settings, such as BCIs. This subspace necessitates automated processing with performance that does not strictly depend on the number of available EEG channels due to the variety of montages used, with the intent of maintaining the required expertise to a minimum.

A key consideration regarding algorithms for ocular artifacts is their public availability and the possibility for immediate use in an analysis pipeline. In recent years, methods based on Wavelet Transform such as [9, 17, 30, 32], Empirical Mode Decomposition such as [43], or even hybrid approaches that combine multiple algorithms in a sequence, a few examples are [28, 29, 44, 50], have been published.

Although the above-mentioned methods reported promising results, only some, but by no means the majority of these methods are readily available in widely-used toolboxes such as EEGLAB [13] for MATLAB and MNE [18] for Python. In the current paper, we will confine ourselves to only considering methods that are available as ready-to-use packages, and have an easy implementation (which does not require extensive user intervention on these platforms. Such tools provide an easily replicable level playing-field for performance comparison and in the most prevalent use.

The methods both EEGLAB and MNE provide can be classified into two broad families, each of which presents significant drawbacks:

• **Regression-based:** These methods compute transmission factors in order to relate the amplitude between one or more reference channels (e.g. electrooculogram (EOG)) and each EEG channel. The correction of artifacts involves the subtraction of the estimated proportion of the EOG from the EEG. Bidirectional contamination limits the accuracy of regression approaches due to their oversimplifying assumption that the EOG channel does not also contain brain electrical activity. Consequently, a simple subtraction may not only remove ocular artifacts but also relevant cerebral activity. In addition, regression-based methods require the presence of a reference channel specifically dedicated to artifact measurement, which sometimes may not be available.
• **Blind source separation (BSS)**: BSS aims to extract the individual unknown source signals (brain signals and artifacts) from the recorded EEG signals which are the result of their mixtures. BSS methods work on the assumption that the number of sources can be, at most, equal to the number of observed channels. Moreover, good performance relies on having a large amount of signal, especially when the channel count is high—this makes the approach more suitable for offline analyses than for online use in BCI systems. Additional assumptions are generally required, e.g. for the most popular family of BSS algorithms, independent component analysis (ICA), the sources need to be non-Gaussian and are assumed to be independent. BSS methods are known for requiring a lot of expertise in the classification of the extracted sources, hence deciding whether or not a source should be removed due to their properties resembling a known source of artifacts, with the final choice being highly subjective from user to user. For this reason, more-recent add-ons to the approach, such as ICLabel [40], use pre-trained classifiers to automate recognition of particular components and reduce the need for human judgment.

In this work, we present a lightweight and easy-to-use method for blink artifact removal from EEG signals using multivariate backward regression, that does not demand large amounts of data. This method is referred to as **A**rtifact **R**eference **M**ultivariate **B**ackward **R**egression (ARMBR). The ARMBR method detects the times at which eye blinks occur, and then estimates their linear scalp projection by regressing a simplified time-locked reference signal against the multi-channel EEG. This then allows the artifacts to be projected out of the signal space, which results in a blink-suppressed EEG set. We validated the performance of the ARMBR method on (a) semi-synthetically generated blink-contaminated EEG signals from 10 participants and (b) two real datasets; the first is obtained from 16 human participants while listening to recordings of natural speech and the second from an ERP-based oddball experiment from 42 human participants. We compare ARMBR’s robustness in suppressing blink artifacts against other commonly used methods in popular EEG analysis toolboxes, such as the signal-space projection (SSP) [18, 45] and the forward-regression method [19] as implemented in the MNE toolbox, the Artifact Subspace Reconstruction method (ASR) [26] as implemented in MNE-compatible Python package [14], and the Infomax ICA coupled with ICLabel [40] as implemented in the EEGLAB toolbox [13]. These methods were selected as being readily available for downloadable and usable with minimal parameter tuning or expert judgment; we also emphasize non-ideal or online use-cases, in which limited training data are available (in this aspect, the ICA-based method is being used somewhat outside its usual scope—but, as we shall see, its performance is nonetheless competitive under some of the conditions).

The comparison we provided, in the case of the semi-synthetic dataset, is in terms of root mean square error (RMSE), SNR, and Pearson correlation ($\rho_\mathrm{pearson}$) between the clean brain wave signals and the blink-suppressed signals. As for the real dataset, since the blink-suppressed ground truth is unavailable, we computed a semi-normalized correlation coefficient, *R*, for blink-epochs and non-blink-epochs between the blink-contaminated EEG signals and the blink-suppressed signals. We also computed relative mean absolute error (RMAE) of power-spectral-density (PSD) features in three frequency bands (8–12 Hz, 12–25 Hz, and 25–40 Hz) that typically contain EEG signals of interest while being less affected by blink artifacts. Lastly, we evaluated whether the blink-suppressed EEG signals retain task-relevant responses from an ERP-based oddball dataset. There, we computed the average ERP before and after blink removal (using ARMBR). When tested on the semi-synthetically generated blink-contaminated EEG signal, ARMBR exhibited smaller RMSE and greater SNR and $\rho_\mathrm{pearson}$ as compared to the two MNE method and comparable (or better in some scenarios) performance to ASR and ICA+IClabel. When tested on the real dataset, ARMBR exhibited comparable *R* levels to the ICA+ICLabel method (when trained on long-enough data segments) and better *R* levels than MNE SSP and MNE Reg methods. As for ASR, *R* levels where higher for non-blink intervals. The same conclusion holds for RMAE values computed from frequency bands that are typically less affected by blink components. As for the ERP-based dataset, we showed that ARMBR, successfully preserved the desired ERP signals while removing the unwanted blink artifacts fully automatically. Additionally, we evaluated ARMBR’s potential in online denoising environments and showed its ability to suppress blinks using pre-trained blink spatial filters.

The rest of the paper is organized as follows. Section 2.1 describes the semi-synthetic signal generation method and section 2.2 describes the real dataset that we employed in this paper. Section 3 presents the steps of the ARMBR method, namely, blink artifact detection (section 3.1) and blink artifact removal (section 3.2). The automated blink selection process is shown in section 4.

The comparison of performance between the ARMBR method and the alternative blink removal methods is reported in section 5. The performance metrics and the alternative blink-removal methods are defined in sections 5.1–5.3, respectively. The quantitative comparison is shown in section 5.4. In section 6, we discuss the ability of ARMBR to be deployed as an online tool for blink artifact denoising. Section 7 highlights ARMBR’s features and provides insight on how to utilize this framework on other artifacts. In section 8, we conclude that the ARMBR method is desirable since (a) it exhibits, in many scenarios, high quantitative performance when compared to alternative blink-removal methods and (b) the required user intervention is limited to identifying the channels that are expected to be heavily influenced by blinks. A Python and MATLAB implementation of the ARMBR method as well as the semi-synthetic and real signals used for validation are available at: https://github.com/S-Shah-Lab/ARMBR.git.

## 2. EEG datasets

To test the performance of ARMBR and compare it to the other tested methods, we employed two strategies: a semi-synthetic-data approach and a real-data approach. For both approaches, we used the corpus of Broderick *et al* [7, 8] in which 19 participants were listening to recordings of natural speech. All signals were collected from a 128-channel ActiveTwo system (BioSemi) at 512 Hz, down-sampled to 128 Hz, and reference to the average of the right and left mastoids. The data were then bandpass-filtered from 1 Hz to 40 Hz using a 4th order Butterworth filter.

In addition, our real-data approach incorporated an event-related potential (ERP) analysis, on data from the OpenNeuro *Nencki-Symfonia EEG/ERP dataset* [16]. This dataset contains real EEG data from 42 participants performing a visual oddball task. In the oddball paradigm, three stimuli (rare target, rare distractor, frequent standard) were presented in a pseudorandom order with the restriction that two rare stimuli could not appear in a row. Participants were instructed to push a button in response to the target stimulus and inhibit responses to other stimuli. The session contained 660 stimuli, which included approximately 12% targets, 12% deviant, and 76% standard stimuli. Each stimulus was presented for 200 ms with a 1200–1600 ms inter-stimulus interval [16]. EEG data were recorded using an actiCHamp amplifier system (Brain Products GmbH, Munich, Germany) and Brain Vision Recorder at a sampling rate of 1000 Hz, from 128 active electrodes (actiCAP, Brain Products, Munich, Germany). More details are provided by Dzianok *et al* [16].

### 2.1. Semi-synthetic-data approach

A frequent problem for artifact correction methods is quantifying the performance, as the ground truth that can be used to assess the accuracy of the correction is not available. To address this issue, we used blink-suppressed EEG signals that were synthetically contaminated with known blink artifacts. We used data from ten participants from the data-set, choosing those subjects for whom blinks were most easily identifiable by eye. From each participant’s data, we identified a 15 s ‘clean’ segment without blink artifacts, and a 15 s segment containing blinks^4^4Only 10 participants (out of 19) were included in the semi-synthetic analysis due to the inability to identify a sufficiently long blink-free segment (15 s) in the remaining data. This limitation did not affect the real data analysis, where all participants were included.. These were then combined using a method inspired by [29]. Each EEG channel was constructed as the sum of two components; clean and blink, denoted $S_\mathrm{clean}$ and $S_\mathrm{blink}$, respectively. The EEG signal of the *j*th channel, denoted *S*_*j*_ with $j \in \{1,\ldots,128\}$, can be written as

\begin{equation*} S_{j} = S_\mathrm{clean}^{\left(j\right)} + S_\mathrm{blink}^{\left(j\right)}.\end{equation*}

**Figure 1.**
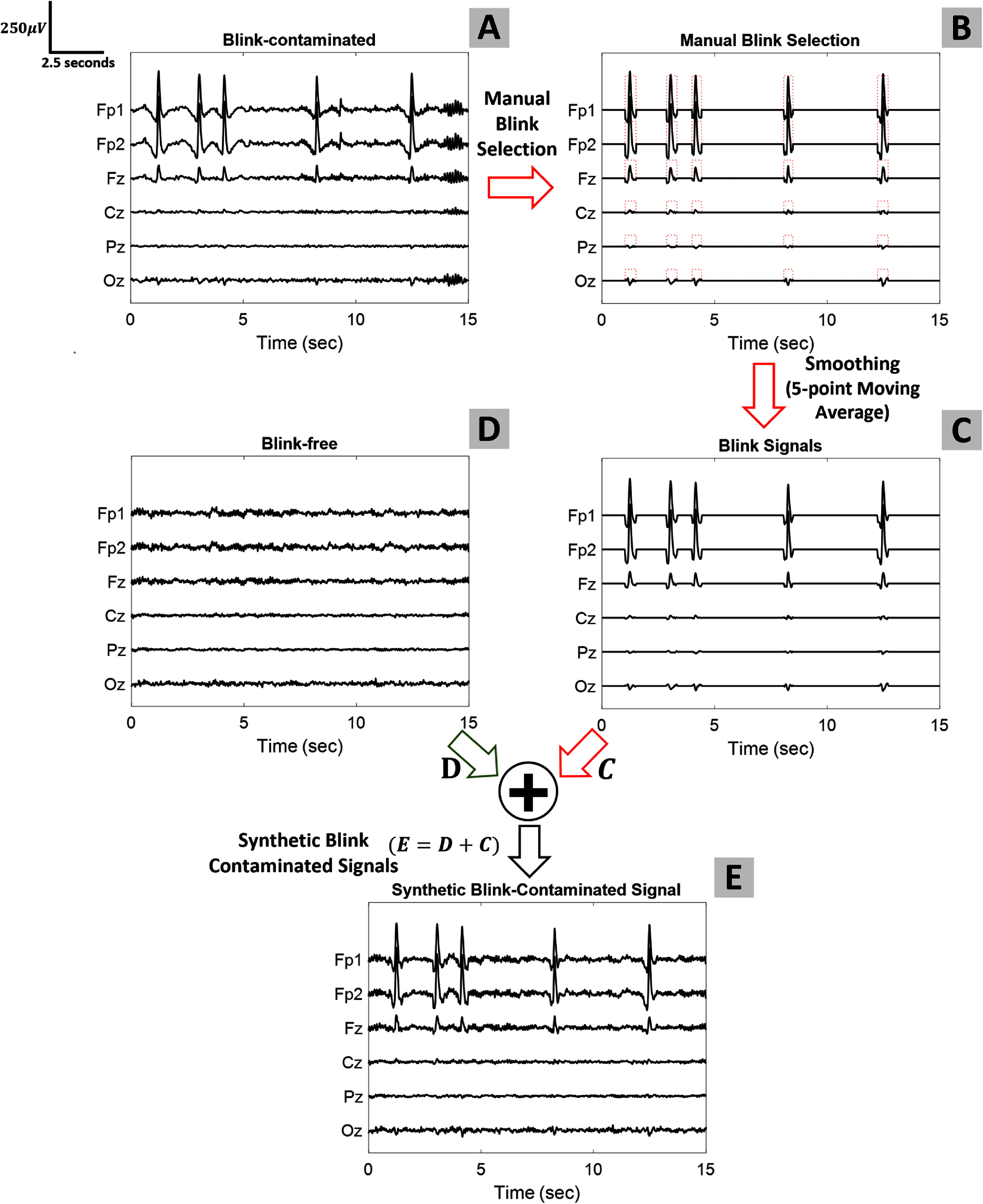
Illustration of the construction of semi-synthetic blink-contaminated EEG. Participant 17 was used for illustration. Subplot (A) shows 15 s of blink-contaminated EEG signals. Subplot (B) shows the blink portions manually labeled and the non-blink regions set to zero. Subplot (C) shows $S_\mathrm{blink}$, the result of applying the MATLAB functionsmooth to B, to attenuate residual non-EOG signals. Subplot (D) shows a different 15 s segment $S_\mathrm{clean}$ from the same subject’s data, chosen to be free of blinks. Subplot (E) shows the sum $S_\mathrm{clean} + S_\mathrm{blink}$ to form the synthetic blink-contaminated signal, *S*. For simplicity, the figure only shows a subset of the channels (Fp1, Fp2, Fz, Cz, Pz, and Oz).

Figure 1 illustrates the process we employed to construct blink-contaminated EEG signals. The signals used in this example are those of participant 17. For simplicity, we only showed channels Fp1, Fp2, Fz, Cz, Pz, and Oz. However, the same process is employed for the full channel set. First, a blink-contaminated region, $S_\mathrm{blink}$, is extracted as following:

(i) We identified a region from the EEG recording contaminated with blinks (illustrated by subplot (A) of figure 1). These regions were filtered from 1 to 15 Hz.
(ii) We manually labeled the blink portions of the signals (red dotted rectangular train in subplot (B) of figure 1). The amplitude of the EEG signal in the blink region is kept the same and the rest of the signal (non-blink regions) is padded with zeros.
(iii) To attenuate the amplitude of the brain-wave residues from the blink signal, we employed the MATLAB functionsmooth (concept adopted from [29])(subplot (C) of figure 1).

Second, $S_\mathrm{clean}$ is extracted from regions of the recording where no blinks were observed (illustrated by subplot (D) of figure 1). The duration of $S_\mathrm{clean}$ and $S_\mathrm{blink}$ obtained from each participant was 15 s.

By adding the blink-suppressed signal, $S_\mathrm{clean}$ (signals in subplot (D) of figure 1), to the blink signal, $S_\mathrm{blink}$ (subplot (C) of figure 1), we obtained the synthetic blink-contaminated EEG signal, $S = S_\mathrm{clean} + S_\mathrm{blink}$ (subplot (E) of figure 1).

This semi-synthetic blink-contaminated EEG generation method is desirable since (a) it provides us with a ground truth clean brain waves that are extracted from real human data, (b) it takes into account the inter-participant blink characteristics, and (c) it automatically modulates the magnitude of the blink component in individual channels relative to their distance to the eyes (such that the blink component is the strongest for channels closer to the eyes). The semi-synthetic blink-contaminated EEG signals are available at the Github repository: https://github.com/S-Shah-Lab/ARMBR.git.

### 2.2. Real-data approach

Drawing from the same publicly available 19-participant natural-language listening dataset [7, 8] that we used in the semi-synthetic data analysis, we also performed a wider-ranging analysis without artificially manipulating the EEG. For each participant, up to 20 three-minute segments were available. First, the data were processed as described above. Bad channels that significantly deviate from the rest^5^5The standard deviation across time points of each channel was computed. Channels whose standard deviation was greater than 4 times, or less than 1/6, the average standard deviation of all channels were flagged. were removed and reconstructed via linear interpolation. In this analysis, we excluded participant 3 since one of the blink reference channels was flagged bad and interpolated, subject 4 since it was difficult to recognize blink artifacts, and subject 5 since the signal’s amplitude was remarkably larger (by a factor of ∼100) than a normal EEG signal. The analysis was carried out on the remaining 16 participants.

For each participant, we concatenated all runs sequentially to form a longer EEG recording. We used the first 10 min of the concatenated EEG recording of each participant, which was then split into two segments; the first 5 min were used for training and the second 5 min were used for testing. In section 5.5, we evaluate the various algorithms’ ability to generalize to unseen data in an online application when trained on varying lengths of data (15 s through 5 min); we refer to this analysis as online analysis. We additionally report the performance of these methods for offline analysis (training and testing on the same data).

In addition, for our ERP analysis (see sections 2 and 5.6), we processed the data following the method of the dataset’s owners [16]: the EEG data were band-pass filtered from 0.5 to 40 Hz (we also down-sampled to 250 Hz at this point), re-referenced to the common average, segmented into epochs starting 200 ms before and ending 1000 ms after stimulus presentation, and baseline-corrected.

## 3. Method

The ARMBR method is designed to detect and characterize the eye blink artifacts present in EEG signals and project them out of the signal space, resulting in a set of blink-suppressed EEG signals. The input to our system is a set of *C* EEG channels (*N* samples each) contaminated by blink artifacts, while the output is the blink-suppressed EEG set. The ARMBR framework comprises two stages; blink artifact detection and blink artifact removal.

### 3.1. Blink artifact detection

**Figure 2.**
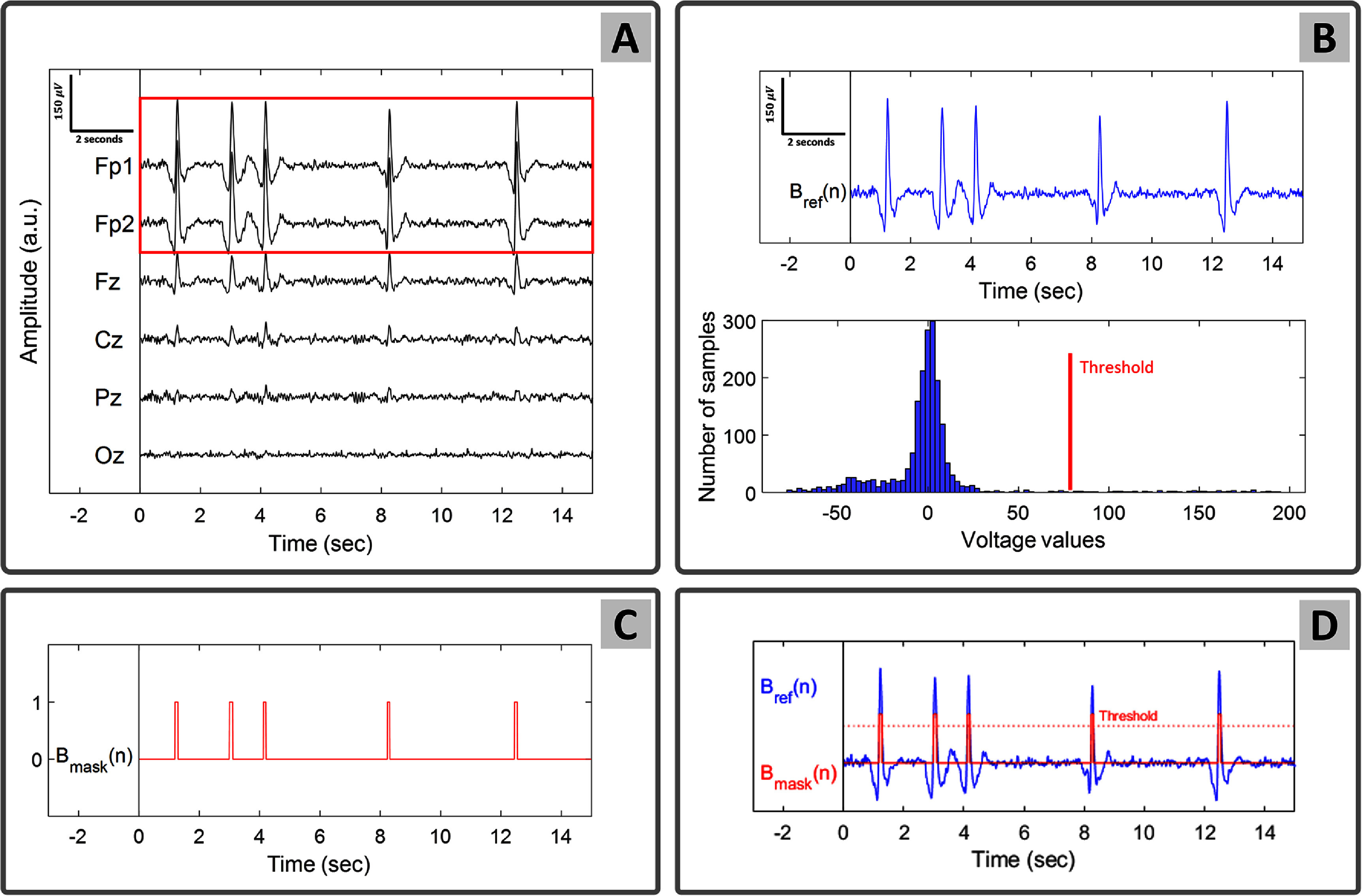
Summary of the blink artifact detection method. Subplot (A) shows the blink-contaminated EEG signals (for simplicity only 6 out of the 128 channels are shown). Fp1 and Fp2 are highlighted as the channels that are most affected by blink artifacts. Subplot (B) shows the blink reference signal which is computed as the average of Fp1 and Fp2, together with a histogram of the resulting voltage values. Subplot (C) shows the blink mask (thresholded version of (B) used as a regression target to estimate the blink spatial filter. Subplot (D) overlays the blink mask onto the blink reference signal.

ARMBR employs an automatic mechanism that detects the blink regions without the need for manual blink selection—which eliminates user intervention. Blink artifacts are detected from a designated subset of EEG channels that are most severely affected by blinks, typically the channels closest to the eyes. In this paper, Fp1 and Fp2 are used as blink-contaminated channels. Figure 2 is an illustration of the blink artifact detection process (the same signals are adopted from figure 1).

First, a blink reference signal, $B_\mathrm{ref}(n) \; | \; n \in [1, N]$ (top blue trace in subplot (B) of figure 2), is constructed by averaging across the time series of all blink-contaminated EEG channels (channels Fp1 and Fp2 in subplot (A) of figure 2). In the typical case, the number of blink reference signals denoted *P*, is equal to 1.

Second, the amplitude of $B_\mathrm{ref}$ is organized in a histogram. The abscissa of this histogram shows voltage values and the ordinate shows the frequency of occurrence of each respective voltage bin. The histogram is illustrated at the bottom of subplot (B) of figure 2.

Third, a blink mask is constructed such that it takes a value of 1 where the voltage of the blink reference signal exceeds a predefined threshold and 0 otherwise. The blink mask can be written as

\begin{align*} B_\mathrm{mask}\left(n\right) = \begin{cases} 1, &amp; \;\mathrm{if}\; B_\mathrm{ref}\left(n\right) > T_0 \\ 0, &amp; \;\mathrm{otherwise}, \end{cases}\end{align*}

where *T*_0_ is the threshold used (subplots (C) and (D) of figure 2). The *T*_0_ value (shown as a red bar in subplots (B) and (D) of figure 2), if desired, could be manually selected based on the user’s judgment, but an alternative method with an automatic selection is presented in section 4.

The blink mask, $B_\mathrm{mask}(n)$, is used for the blink artifacts removal process of section 3.2. Its binarization to values 0 and 1 oversimplifies the blink signal and thereby has a regularizing effect on the regression performed in the next step.

### 3.2. Blink artifact removal

The blink artifact removal starts by considering *X*, a multi-channel time series matrix of dimensions *N* × *C* where *N* is the number of time samples and *C* is the number of EEG channels. The blink mask $B_\mathrm{mask}$ generated in section 3.1 is a *N* × *P* matrix. In most scenarios, including the case considered in this paper, *P* = 1, i.e. a single blink reference signal is distilled down from multiple blink-affected EEG channels by the method described above^6^6Using this projection method for other types of artifact removal may benefit from higher-dimension (*P* > 1) reference signal.. The relationship between *X* and $B_\mathrm{mask}$ is defined by the following system of linear equations:

\begin{align*} X^\star W^\star = B_\mathrm{mask}, \;\;\;\;\;\mbox{where} \;\; X^\star = \left[X,\; \vec{1}\right], \;\;W^{\star\top} = \left[W^{\top},\; \vec{k}\right]\end{align*}

with *W* being the *C* × *P* matrix of *P* spatial filters. A column of ones is concatenated onto the data *X* to allow least-squares estimation of the linear intercept terms $\vec{k} \in \mathcal{R}^P$. By solving the least-squares problem, it is possible to extract $\hat{W}^\star$:

\begin{align*} X^{\star\top} X^\star W &amp; = X^{\star\top} B_\mathrm{mask}\end{align*}

\begin{align*} \hat{W}^\star &amp; = \left(X^{\star\top} X^\star\right)^{-1} X^{\star\top} B_\mathrm{mask}.\end{align*}

From the resulting coefficients, $\hat{W}^\star$, the intercept is removed and the remaining columns rescaled to obtain the spatial filters $\hat{W}$ associated with the given blink reference signals (i.e. the weightings across EEG channels to estimate the target source signals). The rescaling factor is computed such that the spatially-filtered signals $X\hat{W}$ have unit variance. We can then compute the corresponding spatial pattern $\hat{A}$ (i.e. the matrix of linear projection strengths of the source at each EEG electrode position) as follows:

\begin{align*} \hat{A} = \Sigma \hat{W},\end{align*}

where Σ is the *C* × *C* sensor covariance matrix of the multi-channel time series matrix *X* and both $\hat{W}$ and $\hat{A}$ are *C* × *P* matrices. Note that this transformation is valid under the assumption that the source to be removed has zero correlation with the remaining sources [21, 23]. This assumption is also shared by ICA and many other PCA-based methods. The blink artifact spatial component can be estimated by the outer product $\hat{W}\hat{A}^\top$, lastly, given the rescaling applied earlier, it is possible to determine the blink-suppressed spatial component, $M_\mathrm{purge}$, by removing $\hat{W}\hat{A}^\top$ from the identity matrix:

\begin{align*} M_\mathrm{purge} = I - \hat{W}\hat{A}^\top = I - \hat{W}\hat{W}^\top \Sigma^\top = I - \hat{W}\hat{W}^\top \Sigma,\end{align*}

where *I* is the *C* × *C* identity matrix and $\Sigma^\top = \Sigma$ being symmetric.

At this point, the blink artifact removal procedure is completed and the blink-suppressed multi-channel time series matrix, $X_\mathrm{purged}$ can be obtained as:

\begin{align*} X_\mathrm{purged} = X M_\mathrm{purge}\end{align*}

while the blink component is obtained as:

\begin{align*} B_\mathrm{c} = X \hat{W}.\end{align*}

Table 1 summarizes the matrices mentioned in this section and their dimensions.

**Table 1.** Matrix notation used for the blink artifact removal procedure. *N* is the number of each time series data points, while *C* is the number of EEG channels. Note that, for blink removal, we use only one time series, computed as the average of multiple blink-contaminated channels—hence, for purposes of this paper, *P* = 1.

Notation	Explanation	Dimension
*X*	EEG data with blink artifacts	*N* × *C*
$B_\mathrm{ref}$	Time series generated by averaging selected EEG channels	*N* × *P*
$B_\mathrm{mask}$	Mask generated in blink detection	*N* × *P*
$X^\star$	*X* matrix with added column of ones	$N \times (C+1)$
*W*	Spatial filter	*C* × *P*
$\hat{W}^\star$	Resulting coefficients from least-squares solution	$(C+1) \times P$
$\hat{W}$	Spatial filter obtained by solving the system of linear equations	*C* × *P*
Σ	EEG data (*X*) covariance matrix	*C* × *C*
$\hat{A}$	Spatial pattern	*C* × *P*
$M_\mathrm{purge}$	Blink-suppressed spatial component	*C* × *C*
$X_\mathrm{purged}$	Blink-suppressed EEG data (time series without blink artifacts)	*N* × *C*
$B_\mathrm{c}$	Blink component	*N* × *P*

### 3.3. Undesirable artifact removal

Undesirable artifacts, particularly those with a high voltage level, contaminate the EEG signals and could interfere with the blink artifact detection method described in section 3.1. Therefore, we developed a mechanism that detects abrupt voltage jumps or high-voltage artifacts and removes them from the analysis. First, we divide the EEG data into 15 s-long segments. Second, we compute the first derivative of the $B_\mathrm{ref}(n)$ for each segment *s* and denote it $B^{\prime}_\mathrm{ref}(s, n)$. Next, we compute the maximum amplitude of $B^{\prime}_\mathrm{ref}(s, n)$ and denote it $A^s_\mathrm{max}$. A segment *s_r_* is rejected if it satisfies the following condition:

\begin{align*} \Big|A^{s_r}_\mathrm{max} - \mu_{s_p} \Big| > \nu \times \sigma_{s_p},\end{align*}

where $A^{s_r}_\mathrm{max}$ is the maximum voltage of $B^{\prime}_\mathrm{ref}(s_r, n)$, $|.|$ is the absolute value operator, $\mu_{s_p}$ and $\sigma_{s_p}$ are the mean and standard deviation of maximum amplitude values calculated from all previous segments, and *ν* is a constant (we set *ν* = 5 in our work).

## 4. Threshold selection

As previously described, the threshold *T*_0_ can be selected either manually or automatically. An automatic system is desirable since it eliminates the user’s subjective judgment and level of expertise in identifying blink artifacts and setting appropriate levels for blink-artifact selection. We automatically calculate a *T*_0_ level as follows.

First, we ensure that the histogram of $B_\mathrm{ref}(n)$ is skewed to the right, by convention, as we want to focus on the higher values in the long tail of the distribution. If $B_\mathrm{ref}(n)$ is left-skewed, all the values will be multiplied by −1 to make it right-skewed. This choice relies on the fact that when the eyes blink, the eyelid moving across the eye can be interpreted as a varistor that modules the voltage of a dipole (the corneal-retinal potential). This dipole is characterized by a positive voltage at the front of the eyes and a negative voltage at the back, resulting in a positive monophasic deflection of 50–100 *µ*V when recorded above the eye level.

Next, the 0.159 and 0.841 quantiles, *Q*_*a*_ and *Q*_*b*_, are computed for $B_\mathrm{ref}(n)$. For a Gaussian distribution, these quantile values would be equivalent to $\mu \pm \sigma$ (where *µ* and *σ* are the mean and standard deviation, respectively). Additionally, we compute the median, *Q*_2_. Values below *Q*_*a*_ are not considered when detecting blinks—as stated above, we are interested only in the larger positive component. Due to the non-parametric nature of this approach, the statistics are not dramatically affected by isolated single-point large-voltage outliers that could occasionally contaminate the signals. However, this method is negatively affected if such phenomena become more frequent or if prolonged periods of high-voltage levels dominate the EEG signals. To mitigate this, the mechanism proposed in section 3.3 is used.

The considered blink components are then identified by the values greater than the threshold:

\begin{align*} T_0 = Q_{2} + \alpha_0 \times \nu,\end{align*}

where $\nu = (Q_{b} - Q_{a})/2$, and optimal value for *α*_0_ (denoted as $\alpha_0^\star$) is found as follows:

\begin{align*} \alpha_0^\star = \underset{\alpha_0 \; | \; \alpha_0 \in (0, 10]}{\arg\max} \Delta(\alpha_0) \;,\end{align*}

with

**Figure 3.**
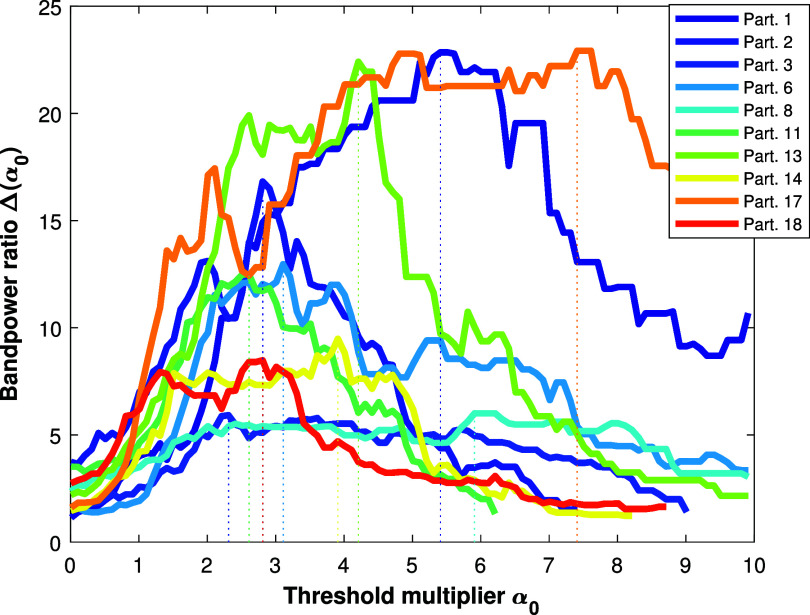
Calculation of optimal threshold multiplier $\alpha_0^\star$ from the blink-contaminated signals of all semi-synthetic data participants. The plot shows the ratio Δ between 1–8 Hz bandpower and 8–40 Hz bandpower of the blink artifact component estimated via ARMBR, as a function of the threshold multiplier from 0 to 10. $\alpha_0^\star$ is chosen as the value that maximizes $\Delta(\alpha_0)$; which is represented by a vertical dashed line.

\begin{align*} \Delta\left(\alpha_0\right) = \frac{\mathbf{E}\left({B^{\alpha_0}_{\mathrm{c},\, lpf}}\right)}{\mathbf{E}\left({B^{\alpha_0}_\mathrm{c} - B^{\alpha_0}_{\mathrm{c},\, lpf}}\right)}\end{align*}

where $\mathbf{E}(.)$ is the energy (sum of the squares of the signal values across time). $B^{\alpha_0}_{\mathrm{c},\, lpf}$ is the blink component $B_\mathrm{c}$ from (9) obtained using ARMBR with threshold *T*_0_ and bandpass filtered (from 1 to 8 Hz). Using (11) and (12), the optimal threshold ($T_0^\star$) is selected. Figure 3 shows the process of calculating $\alpha_0^\star$ (and hence $T_0^\star$) from the blink-contaminated signals for all 10 semi-synthetic participants (for visualization purposes). The average time to compute a threshold based on equation (12) is around 6 s and 3 s for an *α*_0_ step of 0.1 and 0.2, respectively^7^7The results were generated by MATLAB R2023a on a personal computer, with a 12th Gen Intel Core$ ^{\mathrm{TM}}$ i7-1280P CPU running at 1.80 GHz, 32GB RAM, and Windows 11 operating system..

Additionally, we illustrated the effect of the blinking rate on the threshold ($T_0^\star$) estimation on EEG recording of participant 17. The clean signal component, $S_\mathrm{clean}$, is constructed using the same method of section 2.1. The blink component, $S_\mathrm{blink}$, was constructed as follows. First, we isolated and captured the first blink of figure 1 which appears between 1 and 2 s. Second, we concatenated this blink *R* times in order to obtain the desired blinking rate, defined as:

\begin{align*} \mathrm{Blinking\;Rate} = \frac{R}{D}\times60\;{\text{s}}\,{\text{min}}^{-1},\end{align*}

where *D* is the signal’s time duration (15 s in our case) and *R* is a positive integer that identifies the number of blinks over the duration *D*. For instance, assuming *R* = 1, the presence of one blink over the span of 15 s results in a blinking rate of 4 blinks per minute. The blink-contaminated signal is obtained by adding up $S_\mathrm{clean}$ and $S_\mathrm{blink}$.

**Figure 4.**
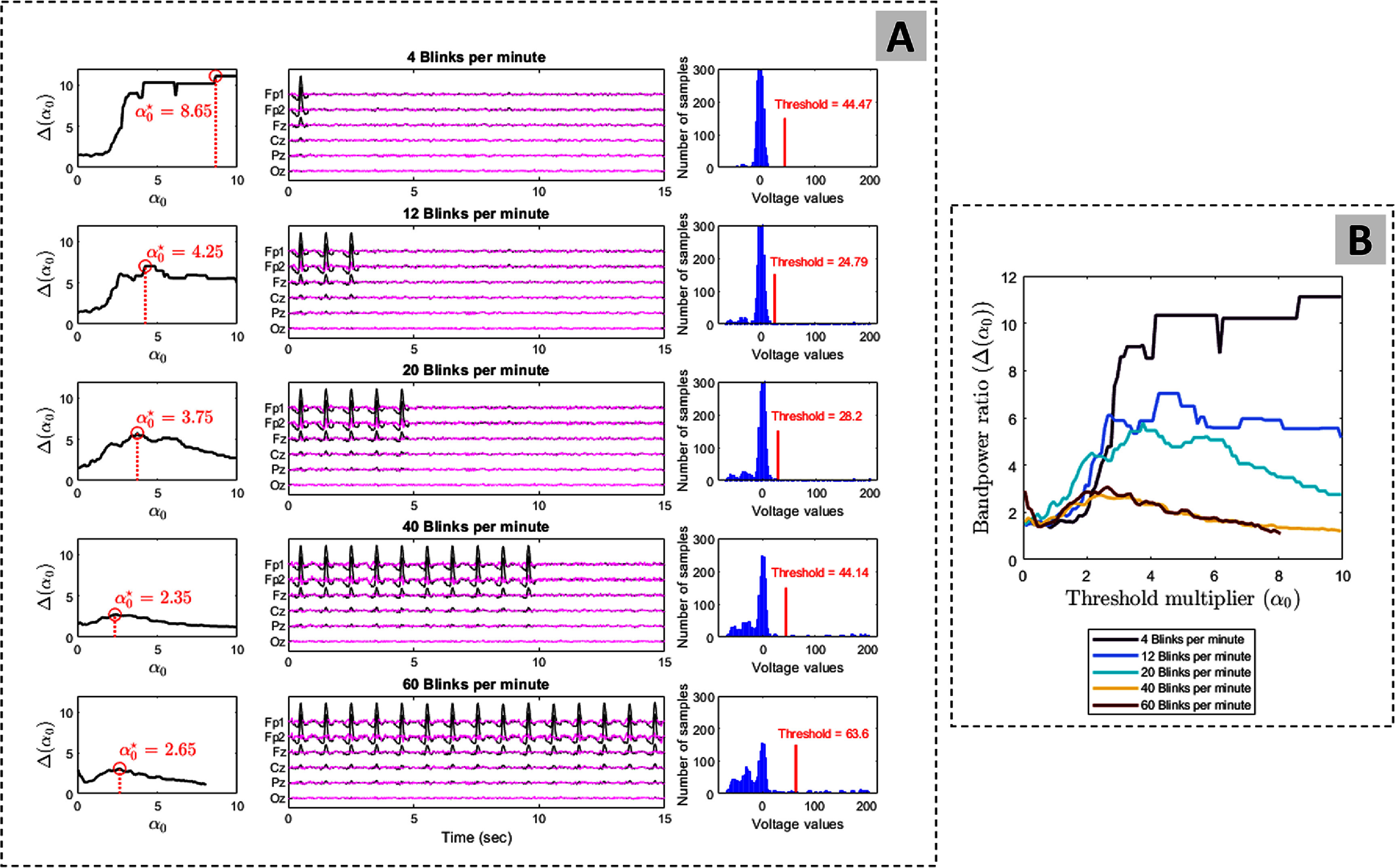
Interaction between threshold multiplier parameter *α*_0_ and the density of blink contamination in semi-synthetic EEG signals. Subplot (A) is divided into five rows showing five different levels of contamination. For each: the left plot shows $\Delta(\alpha_0)$ vs *α*_0_ as in figure 3; the middle plot shows, blink-contaminated (black) against ARMBR-cleaned (magenta) signals; the right plot shows the histogram of voltage values for the blink reference signal, $B_\mathrm{ref}$. The red bar is the optimal threshold ($T_0^\star$) that corresponds to $\alpha_0^\star$, the *α*_0_ value for which Δ is highest. Subplot (B) summarized the $\Delta(\alpha_0)$ vs *α*_0_ graphs for all various blinking rates.

We show in figure 4 the performance of ARMBR for various blinking rates (4, 12, 20, 40, and 60 blinks per minute). The left plots of subplot (A) of figure 4 show $\Delta(\alpha_0)$ vs *α*_0_. In the middle plots of subplot (A) of figure 4, the black and magenta traces correspond to the blink-contaminated and the ARMBR-cleaned EEG signals. The right plots are the histogram of voltage values for the blink reference signal, $B_\mathrm{ref}$, in $\mu V$. The red bar is the optimal threshold ($T_0^\star$) that corresponds to the optimal *α* ($\alpha_0^\star$). Subplot (B) of figure 4 summarized the $\Delta(\alpha_0)$ vs *α*_0_ graphs for all various blinking rates. We can see from figure 4 that ARMBR successfully identified and removed blink artifacts (even when few blinks are present), and the optimal threshold, $T_0^\star$, varied between $T_0^\star \approx 24$ to $T_0^\star \approx 64$^8^8The example in figure 4, based on Participant 17, was used to illustrate the impact of blink rate on ARMBR performance. The optimal threshold value, $T_0^\star$, is typically determined individually for each participant..

## 5. Results

### 5.1. Semi-synthetic dataset performance metrics

To assess and quantify the performance of the proposed method, we employ three metrics, namely, RMSE (15), SNR (16), and Pearson correlation ($\rho_\mathrm{pearson}$) [4] between the clean brain wave signals ($S_\mathrm{clean}$ of figure 1) and the blink-suppressed signals ($X_\mathrm{purged}$ of (8)). For channel *j*, RMSE and SNR can be written as:

\begin{align*} \mathrm{RMSE}^{\left(j\right)} = \sqrt{\frac{1}{N} \sum_{n = 1}^{N} \left(S_\mathrm{clean}^{\left(j\right)}\left(n\right) - X_\mathrm{purged}^{\left(j\right)}\left(n\right)\right)^2}\end{align*}

and

\begin{align*} \mathrm{SNR}^{\left(j\right)} = 10 \mathrm{log}_{10} \left(\frac{\mathrm{STD}\left(S_\mathrm{clean}^{\left(j\right)} \right)}{\mathrm{STD}\left(S_\mathrm{clean}^{\left(j\right)} - X_\mathrm{purged}^{\left(j\right)}\right)} \right)\end{align*}

where *N* is the number of data points and STD is the standard deviation. Low RMSE values (closer to zero) and high SNR and $\rho_\mathrm{pearson}$ values (closer to 1) indicate successful artifact removal.

### 5.2. Real dataset performance metrics

In the case of real datasets, the blink-suppressed ground truth EEG signals are unknown. Therefore, the above-mentioned metrics can not be used. Instead, we define blink and non-blink epochs to evaluate performance as follows. To identify candidate blink intervals without biasing the evaluation in favor of ARMBR, we use the MNE functionfind_eog_events, which detects peaks in the EOG signal and marks ±500 ms around each peak as potential blink intervals. These candidate intervals are then visually inspected by an expert to ensure accurate identification of true blink events and the exclusion of unrelated artifacts. Non-blink-epochs are extracted from time periods before or after the blink epochs occur. Both types of epochs have the same trial number and are of length 1 s. All epochs are visually inspected by an expert. We compute a semi-normalized correlation coefficient, *R*, for the blink-epochs and the non-blink-epochs between the blink-contaminated signals and the blink-suppressed signals after applying a blink removal method. *R* is defined as:

\begin{align*} R = \frac{\mathrm{Cov}\left(X, X_\mathrm{purged}\right)}{\mathrm{Var}\left(X\right)},\end{align*}

where $\mathrm{Cov}(.)$ denotes the covariance between the two signals, and $\mathrm{Var}(.)$ denotes variance.

**Figure 5.**
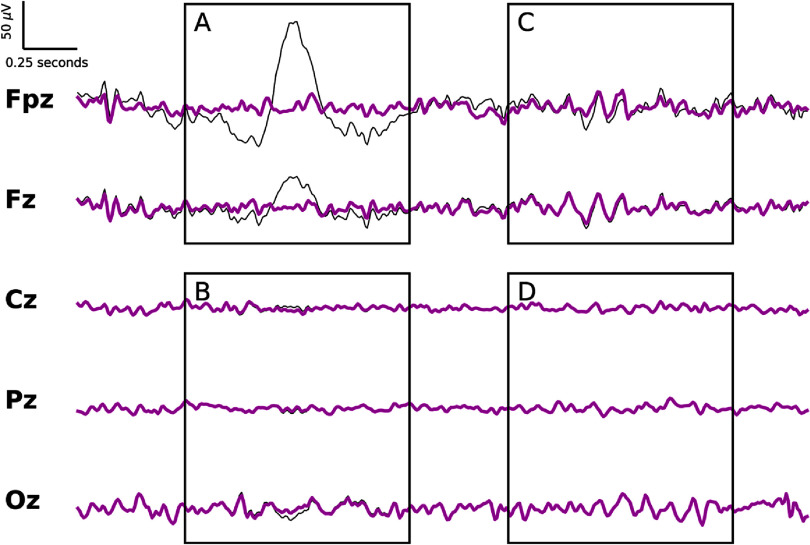
Blink-contaminated (black) and ARMBR-cleaned (magenta) signals from the real data from participant 1. Boxes labeled A through D illustrate four different segments in our data: A shows a blink epoch in blink-affect channels; B shows the same blink epoch in less-affected channels; C shows a non-blink epoch in blink-affect channels; D shows the same non-blink epoch in blink less-affected channels. Semi-normalized correlation coefficients *R* are computed between the contaminated and cleaned signals in each of these regions, to provide insight into algorithm performance (we expect *R* close to +1 in regions B, C and D, and low positive values of *R* in region (A).

We show in figure 5 an illustration of blink (regions A and B) and non-blink epochs (regions C and D) on blink affected (regions A and C) and less-affected (regions B and D) channels. After blink removal, *R* is supposed to be around +1 for non-blink-epochs computed from all channels (regions C and D of figure 5); which indicates that the blink removal method did not remove desired non-blink components from the signal. Additionally, *R* is supposed to be around +1 for blink-epochs computed from blink less-affected channels (region B of figure 5). On the other hand, *R* is supposed to be low for blink-epochs computed from blink-affected channels (region A of figure 5), indicating successful removal of most of the variance of the blink epoch (presumably attributable to the blink artifact itself). When we obtain a very high *R* (around +1) for blink-epochs computed from blink-affected channels, after applying the blink removal method, this indicates that the method failed to remove the blink artifacts.

Additionally, we computed the RMAE of the PSD to assess the effect of artifact removal on three frequency bands—8–12 Hz, 12–25 Hz, and 25 to −40 Hz—that frequently contain EEG signal components of interest while overlapping relatively little with blink artifacts. The RMAE, inspired by Maddirala and Veluvolu [28], was computed as follows:

\begin{align*} \mathrm{RMAE} = \frac{1}{f-l} \sum_{f = l}^j \bigg| \frac{P_{X}\left(f\,\right) - P_{X_\mathrm{purged}}\left(f\,\right)}{P_{X}\left(f\,\right)}\bigg|,\end{align*}

where *l* and *j* represent the indices of the first and last frequencies of a specific band, $P_{X}(f)$ and $P_{X_\mathrm{purged}}(f)$ are the power spectra of the blink-contaminated and the blink-suppressed EEG signals, respectively.

### 5.3. Alternative methods

We compare the performance of our proposed method to other widely used methods in EEGLAB and MNE. These methods are:

(i) MNE SSP [35]: SSP is a technique for removing noise from EEG and MEG signals by projecting the signal onto a lower-dimensional subspace orthogonal to the noise direction [35, 45]. Blink artifact removal is achieved by computing SSP projectors from EOG signals, using the class methodmne.preprocessing.compute$\_$proj$\_$eog [36]. The number of artifact projectors is selected by the user, which requires some level of skill and expertise.
(ii) MNE regression [34]: This method uses forward regression to remove artifacts as described in [10, 19, 34]. The regression coefficients are the results of a linear relationship between each EEG data and the EOG data. The blink artifacts are removed by subtracting the weighted EOG signal (multiplied by the regression coefficients) from the EEG signal [34].
(iii) ASR [26]: ASR is an automated, online or offline component-based artifact removal method for removing transient or large-amplitude artifacts in multi-channel EEG recordings. It repeatedly computes a principal component analysis (PCA) on covariance matrices to detect artifacts based on their statistical properties in the component subspace [6, 14, 26]. In this work, we used the MNE-compatible Python implementation of ASRasrpy [14].
(iv) EEGLAB’s Infomax ICA [3, 31] followed by ICLabel [40]: The ICA method is a statistical-based filter that has been successfully employed to remove eye-blink artifacts from the multichannel EEG signals [29]. There are numerous methods in the literature that employ ICA for blink removal. In this paper, we focus on the Infomax method, shown to perform well in separating the independent sources in EEG signals [24]. The algorithm maximizes the information flow through the network by adjusting the weights of the demixing matrix [27]. The blink IC identification is then achieved using ICLabel; an automated EEG IC classifier that was shown to perform better and faster than other publicly available EEG IC classifiers [40]. ICLabel computes IC class probabilities across 7 IC classes, namely, ‘Brain ICs,’ ‘Muscle ICs,’ ‘Eye ICs,’ ‘Heart ICs,’ ‘Line Noise,’ ‘Channel Noise,’ and ‘Other ICs.’ This method presents a solution to reduce reliance on the user’s expert judgment and level of expertise. Here, ICs are first computed using EEGLAB Infomax. ICLabel is then applied and only ‘Eye ICs’ with a confidence of 90% and higher were excluded, while all remaining ICs were used to reconstruct the EEG signals.

Table 2 summarized the level of user intervention for ARMBR as well as the alternative above-mentioned blink-removal methods.

**Table 2.** Level of user intervention for the considered algorithms.

Method	User Intervention
MNE SSP	**(Moderate)**. Identify blink source channels and specify the number of blink components to be removed.
MNE Reg	**(Minimal)**. Identify blink source channels.
ASR	**(Minimal)**. Choose a standard deviation cutoff for rejection (preset in theasrpy implementation).
EEGLAB ICA Infomax + ICLabel	**(Minimal)**. Choose a confidence threshold beyond which ICs are marked for exclusion.
ARMBR	**(Minimal)**. Identify a set of channels that are mostly influenced by blink artifacts.

### 5.4. Comparison on semi-synthetic signals

**Figure 6.**
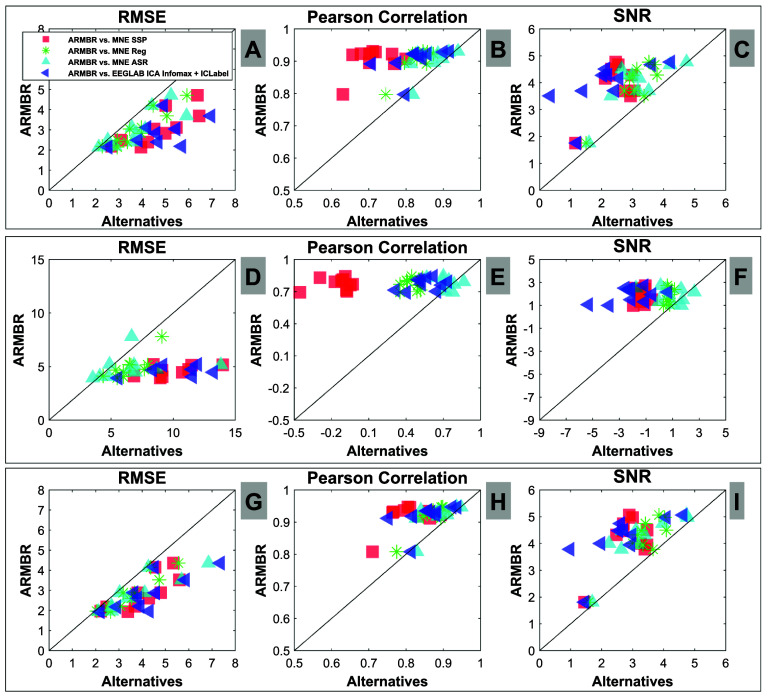
Comparison using three different error metrics (RMSE, SNR, and $\rho_\mathrm{pearson}$) between the ARMBR method and each of the alternative methods, (MNE SSP shown as red squares, MNE Reg shown as green stars, ASR as cyan triangles, and EEGLAB Infomax ICA + ICLabel shown as blue triangles). Results are shown separately for all channels (top row), blink-affected channels only (middle row), and less-affected channels only (bottom row). The diagonal line represents the locus along which both methods exhibit equal performance.

We compute RMSE, SNR, and $\rho_\mathrm{pearson}$ for our proposed method as well as the 4 alternative methods of section 5.3 for (a) all channels, (b) the channels most affected by blinks, and (c) the channels least affected by blinks^9^9In the labeling scheme of the ABC sensor net, the channels affected by blinks the most were channels C8 (AF8), C9, C14, C15 (approximately AF4), C16 (Fp2), C17 (Fpz), C18, C19 (AFz), C27, C28 (approximately AF3), C29 (Fp1), C30 (AF7), and C31. The rest were considered less affected.. These results are reported in the scatter plots of figure 6. Here, each point represents the average metric for a specific participant, 10 participants in total^10^10Tables A1–A3 of the appendix show the RMSE, SNR, and $\rho_\mathrm{pearson}$ for our proposed method as well as the 4 alternative methods of for (a) all channels, (b) the channels most affected by blinks, and (c) the channels least affected by blinks, respectively, for each participant.. The comparisons involve ARMBR with the different methods: MNE SSP (red squares), MNE Reg (green stars), ASR (cyan triangles) and EEGLAB ICA Infomax followed by ICLabel (blue triangles). The diagonal line represents the locus along which both methods exhibit the same performance. It is evident from figure 6 that ARMBR yields a consistently lower RMSE when compared to MNE SSP, MNE Reg, and Infomax + ICLabel when RMSE was computed from all channels (subplot (A)), blink-affected channels (subplot (D)), and blink less-affected channels (subplot (G)). ASR yields comparable RMSE to ARMBR, especially for blink-affected channels.

Accordingly, the Pearson correlation metric (subplots (B), (E) and (H) of figure 6) and SNR metric (subplots (C), (F) and (I) of figure 6) are consistently higher for ARMBR than for MNE SSP, MNE Reg, and Infomax + ICLabel. Similar to RMSE, ASR yields comparable SNR and Pearson correlation levels to ARMBR, especially for blink-affected channels. Note that for some participants, the Pearson correlation metric calculated for the MNE SSP exhibited negative values for the blink-affected channels. In a two-sided Wilcoxon signed rank test [46], the advantage of ARMBR over each alternative method in each channel subset was found to be statistically significant (*p* < 0.05 for all tests, reported in table 3). Note that the results remain significant when we used the adjusted significant level $\alpha^\star$ obtained using the Bonferroni correction [2, 39] (except when testing the Pearson correlation between ARMBR and Infomax for all channels). Based on the Bonferroni correction, $\alpha^\star = \alpha / T$ where *T* is the different performed test groups. In our case, *T* = 4 which results in $\alpha^\star = 0.0125$. Additionally, we conducted 3 separate one-way Analysis of Variance (ANOVA) to compare the mean across all five blink removal methods, in terms of the RMSE, SNR, and Pearson correlation. Statistical significance was assessed for *α* = 0.05. We obtain the following results; RMSE: *F* = 4.19, *p*-value = 0.0057, SNR: *F* = 6.27, *p*-value$ < $0.001, Pearson correction: *F* = 23.79, *p*-value$ < $0.001.

**Table 3.** *P*-values resulting from two-sided Wilcoxon signed rank tests of each performance metric on semi-synthetic EEG data, between ARMBR and each competing algorithm.

	All Channels	Blink-Affected Channels	Blink Less-Affected Channels
RMSE	A: *p*-value = 0.002 B: *p*-value = 0.002 C: *p*-value = 0.002 D: *p*-value = 0.004	A: *p*-value = 0.002 B: *p*-value = 0.002 C: *p*-value = 0.002 D: *p*-value = 0.106	A: *p*-value = 0.002 B: *p*-value = 0.002 C: *p*-value = 0.002 D: *p*-value = 0.002
SNR	A: *p*-value = 0.002 B: *p*-value = 0.002 C: *p*-value = 0.002 D: *p*-value = 0.002	A: *p*-value = 0.002 B: *p*-value = 0.002 C: *p*-value = 0.002 D: *p*-value = 0.065	A: *p*-value = 0.002 B: *p*-value = 0.002 C: *p*-value = 0.002 D: *p*-value = 0.002
Pearson correlation	A: *p*-value = 0.002 B: *p*-value = 0.002 C: *p*-value = 0.004 D: *p*-value = 0.027	A: *p*-value = 0.002 B: *p*-value = 0.002 C: *p*-value = 0.002 D: *p*-value = 0.275	A: *p*-value = 0.002 B: *p*-value = 0.002 C: *p*-value = 0.004 D: *p*-value = 0.020

A: ARMBR vs MNE SSP.

B: ARMBR vs MNE Reg.

C: ARMBR vs EEGLAB ICA Infomax + ICLabel.

D: ARMBR vs ASR.

**Figure 7.**
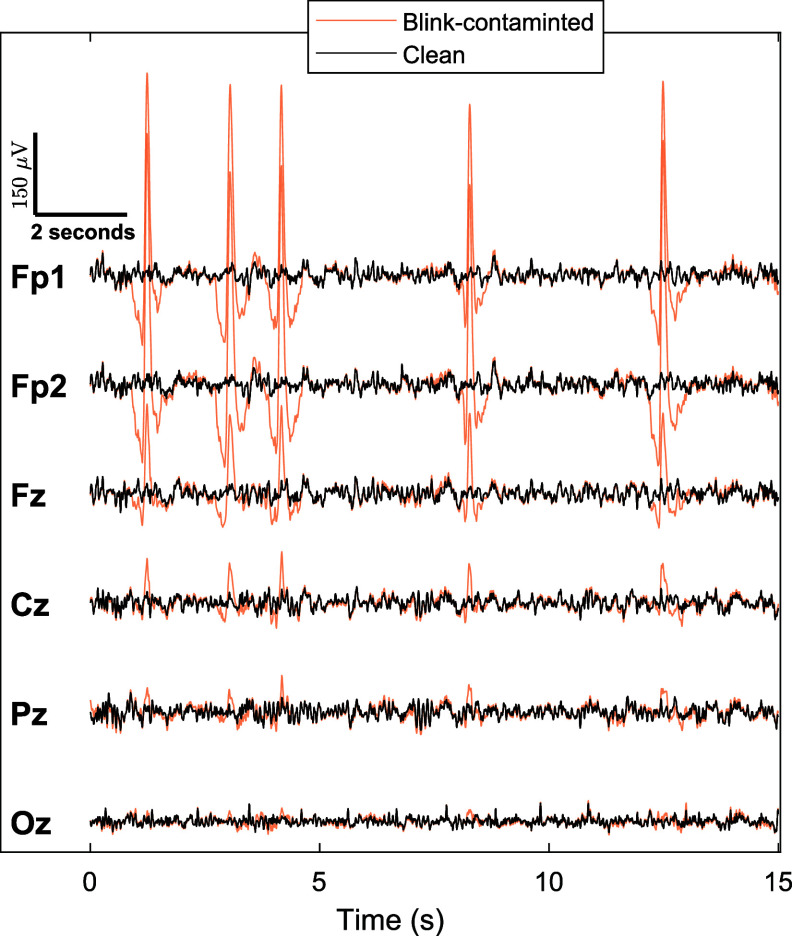
Example segment from the semi-synthetic EEG data-set for participant 17, showing the clean signals (known ground truth) and blink-contaminated composite signals (algorithm inputs).For clarity only 6 channels (Fp1, Fp2, Fz, Cz, Pz, and Oz) are shown out of the actual 128.

**Figure 8.**
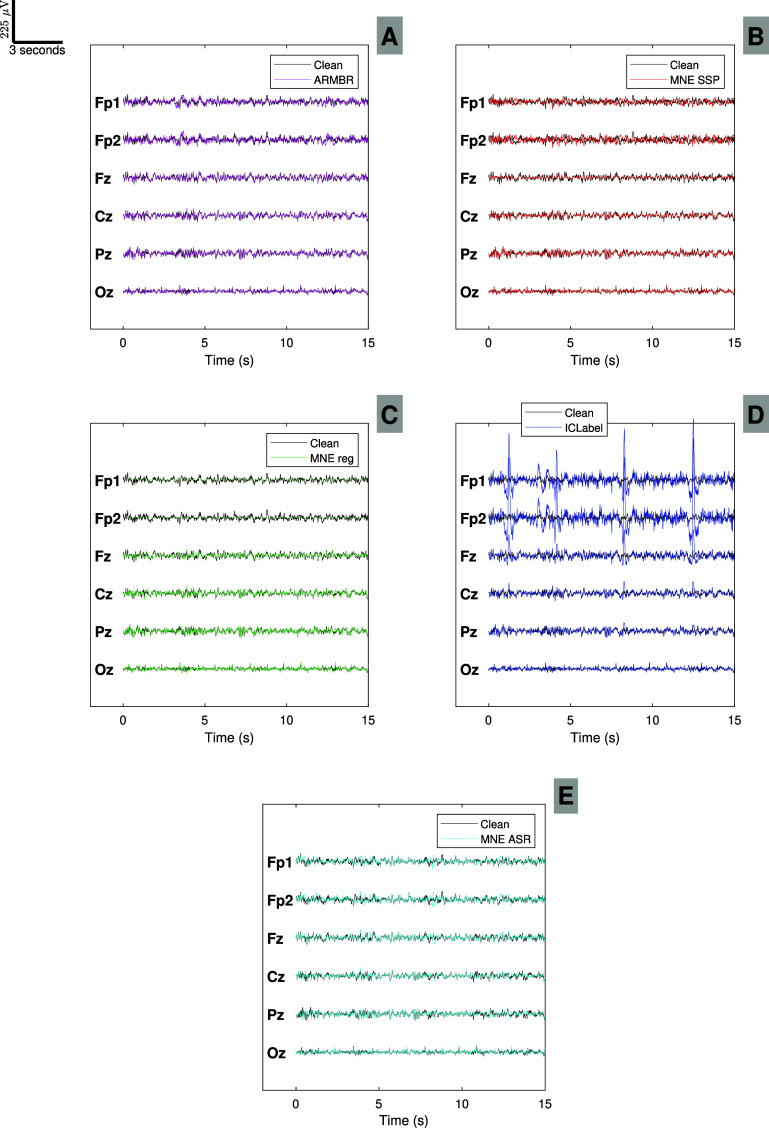
Blink artifact removal performance on an example segment of semi-synthetic data for participant 17 (for simplicity, only 6 of the 128 channels are shown). Subplot (A) shows ARMBR, subplot (B) shows MNE SSP, subplot (C) shows MNE Reg, subplot (D) shows EEGLAB ICA Infomax + ICLabel, and subplot (E) shows ASR. Black traces show the clean ground-truth signal, whereas the overlaid color traces show algorithm outputs. Note that, in our synthetic-data tests, the algorithms were trained on only 15 s of data—the very small ratio of time-points to channels likely explains the failure of blink removal via ICA, an algorithm from which we expect good performance when the ratio is larger.

Moreover, we show in figure 7 the EEG recording of participant 17 from channels Fp1, Fp2, Fz, Cz, Pz, and Oz (we use this reduced set of channels for simplicity). The black and orange traces in figure 7 represent the clean and blink-contaminated EEG signals, respectively. Fp1 and Fp2 clearly show the most prominent blink artifacts, therefore they have been used as blink references for the ARMBR, MNE SSP, and MNE Reg. As we move farther from the eyes, the blink effect attenuates (e.g. at Fz). This effect results in negligible components in even farther channels (Cz, Pz, and Oz). We apply ARMBR, MNE SSP, MNE Reg, ASR, and EEGLAB ICA Infomax + ICLabel to remove the blink artifacts, and the results are shown in figure 8. These methods were applied to all 128 EEG channel recordings of a duration of 15 s.

**Figure A11.**
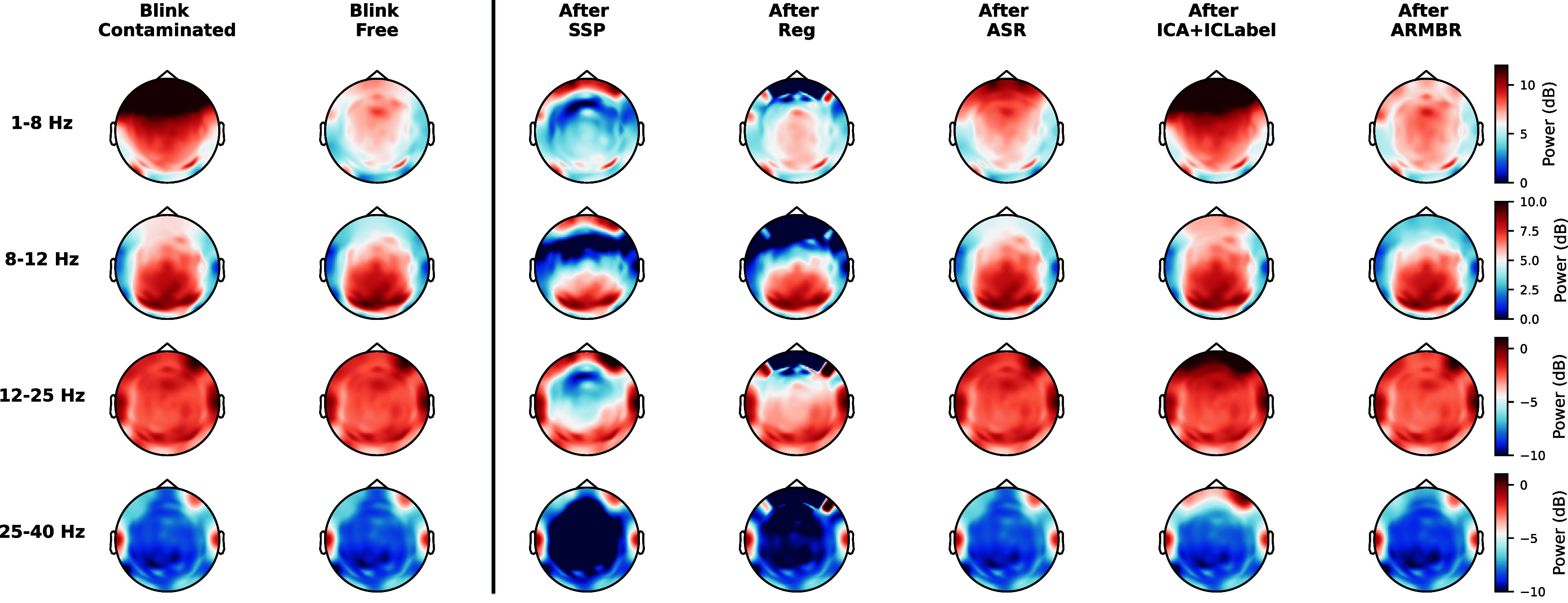
Group-averaged scalp topographies for each blink artifact removal method across three frequency bands: 1–8 Hz, 8–12 Hz, and 12–40 Hz. Each row corresponds to a frequency band. The first and second columns represent the blink-contaminated and blink-free signals, respectively. The remaining columns show the results of the different artifact removal methods applied to the blink-contaminated EEG data. Color indicates power in decibels (dB), averaged across all participants.

Figure 8 subplot (A) shows the contaminated EEG signals after applying the ARMBR method in magenta, with the clean (ground-truth) signal in black. The blink-suppressed signals visually match the clean signals. This is observed for channels strongly affected by blink components (Fp1, Fp2, and Fz) as well as channels with negligible effects (Cz, Pz, and Oz). The red time series in subplot (B) of figure 8 shows the results of the MNE SSP method. It appears that the blink-suppressed signals obtained using MNE SSP exhibit less resemblance to the ground truth compared to those obtained using ARMBR. In subplot (C) of figure 8, the outcomes of the MNE Reg method are highlighted in green. This technique transforms the Fp1 and Fp2 channels into straight lines, as expected. This occurs because the regression approach calculates coefficients to scale the blink reference signals prior to their subtraction from the target signal. In this case, employing dedicated EOG channels as blink references rather than EEG channels would be advisable to avoid reducing the rank of the initial EEG signals. In subplot (D) of figure 8, the blue traces represent the results from EEGLAB ICA Infomax + ICLabel. In this specific case, ICLabel did not identify any blink ICs for the participant, leading to the persistence of blink artifacts in the final signals. It is important to mention that the performance of Infomax + ICLabel was highly variable, primarily due to the relatively short data segments and the use of an automatic IC classification approach, which occasionally failed to assign a high-confidence label ($\unicode{x2A7E}90\%$) to any component. This limitation is discussed further in the Limitations section. Comparison plots of blink removal methods for all 10 participants are available in appendix. Lastly, in subplot (E) of figure 8, the cyan traces represent the results from ASR method. ASR method yielded comparable results to the ARMBR method. Additionally, figure A11 in appendix presents scalp topographies of PSD, averaged across all semi-synthetic data participants for each method. These topographies are shown separately for the 1–8 Hz band (typically most affected by blink artifacts) and the 8–12 Hz, 12–25 Hz, and 25–40 Hz bands (typically less affected). The scalp maps reflect spatial distributions of PSD changes before and after artifact removal.

**Figure 9.**
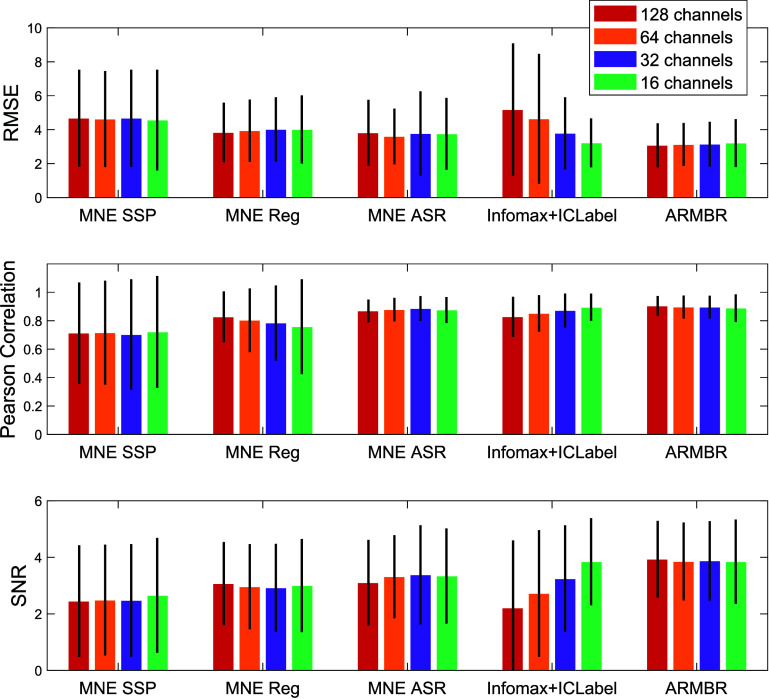
Sensitivity to channel count in overall performance on synthetic data sets. Performance of ARMBR, MNE SSP, MNE reg, ASR, and EEGLAB Infomax ICA + ICLabel for the original 128-channel montage (red bars) and three subsets of this montage—specifically, montages with 64 (orange bars), 32 (purple bars), and 16 channels (green bars). The performance was reported in terms of RMSE (top subplot), Pearson correlation (middle subplot), and SNR (bottom subplot). Each color bar corresponds to the performance metric’s average of all ten participants. The black vertical line corresponds to the performance metric’s standard deviation across the 10 participants.

Moreover, we tested ARMBR and the other blink-removal methods on three various channel subsets derived from the 128-channel original montage. These subsets follow the layout reported in [5] for 64-channel, 32-channel, and 16-channel montages. The results are reported in figure 9. The bars of figure 9 are obtained as follows. First, we compute an average of the performance metric (RMSE, Pearson correlation, or SNR) over all channels for each participant. Each color bar corresponds to the performance metric’s average, while the black vertical line corresponds to the performance metric’s standard deviation of all 10 participants. We observed that the Infomax-based methods benefited from decreasing the number of channels as RMSE decreased, and both Pearson correlation and SNR increased. This was not the case for ARMBR, MNE SSP, MNE Reg, and ASR. In conclusion, ARMBR yielded the lowest RMSE and the highest Pearson correlation and SNR among the tested methods, with performance closely matching that of the ASR method. These results were consistent across most comparisons, although Infomax-based methods performed comparably in the 16-channel montage configuration.

### 5.5. Comparison on real signals

**Figure 10.**
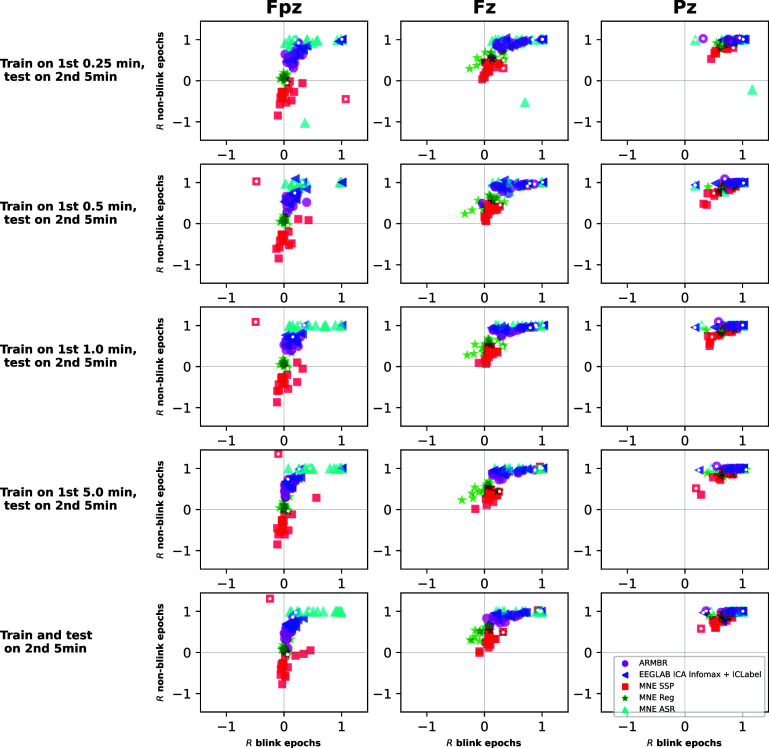
Performance on real data, where the ground truth of the ideal cleaned signal is unknown. Each scatter-plot show the semi-normalized correlation coefficient, *R* between EEG signals before and after cleaning, using the variance of the original (blink-contaminated) signal as the normalizer. The abscissa of each plot shows *R* computed within blink epochs, and the ordinate shows *R* for non-blink epochs. Each point represents a specific participant. Magenta circles denote ARMBR, red squares denote MNE-SSP, green stars denote MNE Reg, cyan triangles denote ASR, and blue triangles denote EEGLAB Infomax ICA + ICLabel. The three columns of subplots show respective performance on three electrodes, at increasing distances from the eyes and their decreasing degrees of blink contamination. The five rows of subplots show respective performance in five different training/testing regimes: in all cases, performance is evaluated on each participant’s second 5 min data segment. The first four rows show performance when training on increasing amounts of data from each participant’s *first* 5 min segment (disjoint from the unseen testing segment), whereas the last row shows performance when training and testing on exactly the same data.

In the case of real data, the blink-free ground truth is unavailable. To gauge the effectiveness of blink removal (figure 10), we employed the semi-normalized correlation coefficient, *R* (defined in equation (17), and compared *R* values between blink-contaminated and blink-suppressed signals in blink epochs and non-blink epochs. Values of *R* close to +1 indicate minimal change to the signal, which we might expect in non-blink epochs (boxes C and D of figure 5, ordinates of figure 10), and also in blink epochs for channels that are largely unaffected by blinks (box B of figure 5, abscissae of subplots to the right of figure 10). Smaller positive values are expected in epochs and channels where a large blink artifact has been removed (box A of figure 5, abscissae of subplots to the left of figure 10). Results are shown in figure 10. ARMBR and Infomax ICA + ICLabel generally perform similarly to each other, and outperform the MNE methods, SSP and Reg. Both the MNE methods tend to over-remove content and change the signal even outside of blink epochs. ASR yields high *R* values for almost all non-blink epochs and most blink epochs. The high *R* values in non-blink epochs reflects ASR’s ability to preserve artifact-free segments. However, similarly high *R* values in blink epochs suggest limited effectiveness in attenuating blink-related artifacts. This observation is further examined in the Limitations section. For a few participants, ICA failed to remove blinks, resulting in high *R* even for Fpz in blink epochs. This effect was more frequent the smaller the data-set, though it persisted for two subjects even when trained on 5 min of data; the effect was not observed in purely post-hoc offline analysis (training and testing on the same data). ARMBR exhibited more consistent values of *R* across subjects and training conditions—even for small training signal segments (consistent with our observations from the previous analysis with semi-synthetic signals). It is also shown in figure 10 that most methods, including ARMBR, undesirably yield low *R* values for non-blink epochs for channel Fpz (which is typically heavily affected by blinks artifacts). This result suggests that in addition to blinks, the methods removed desired non-blink artifact component from the EEG signal.

One participant, number 9, was unusual in that blinks appeared to contaminate every channel of the EEG. This participant’s results are highlighted with a white dot in figure 10. The phenomenon caused erratic results for the SSP method at smaller training-set sizes (red squares in top two Fpz subplots). It also caused both ARMBR and ICA to yield smaller blink-epoch *R* values in channels (such as Pz) that are normally not expected to be blink-contaminated—given that Pz actually *was* blink-contaminated, this result is appropriate.

The differences between algorithms are most obvious in the most blink-affected channel when the training set is smallest—in other words, the top-left subplot of figure 10. ICA+ICLabel outperforms the MNE methods in most cases: the blue triangles are higher than the red squares and green stars in the vertical dimension, indicating that ICA did less collateral damage to non-blink epochs than the other methods. However, the small size of the training data has the expected negative impact on ICA’s stability and robustness: in some cases the blue triangles go to the extreme right of the plot, indicating that ICA+IClabel has completely failed to remove blinks in some subjects. ARMBR performs more stably despite the very low amount of training data: the magenta circles overlap with the non-outlier ICA triangles without throwing out any outlier circles of their own. ASR shows minimal impact on non-blink epochs, as evidenced by its consistently high *R* values.

**Figure 11.**
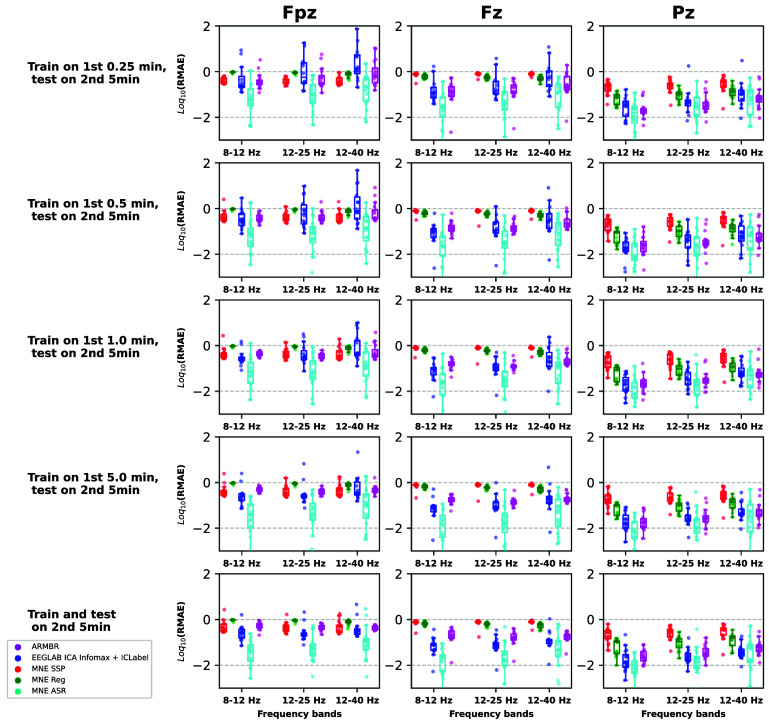
Comparison using RMAE between the ARMBR method and each of the alternative methods, (MNE SSP shown in red, MNE Reg shown in green, ASR shown in cyan, and EEGLAB Infomax ICA + ICLabel shown in blue). Results are shown separately for all channels (top row), blink-affected channels only (middle row), and less-affected channels only (bottom row). The three columns of subplots show respective performance on three electrodes, at increasing distances from the eyes and their decreasing degrees of blink contamination. The five rows of subplots show respective performance in five different training/ testing regimes: in all cases, performance is evaluated on each participant’s second 5 min data segment. The first four rows show performance when training on increasing amounts of data from each participant’s *first* 5 min segment (disjoint from the unseen testing segment), whereas the last row shows performance when training and testing on exactly the same data.

Moreover, we show in figure 11, the RMAE values (equation (18)) of the PSD features computed from three frequency bands – 8–12 Hz, 12–25 Hz, and 25–40 Hz—that frequently contain EEG signal components of interest while overlapping relatively little with blink artifacts. Box plots represent the distribution of the RMAE values across participants (individual participants’ results are also shown as individual symbols). Data points from participants where the blink-removal method failed to remove blinks were not included in this analysis to avoid confusion (an RMAE of 0 is obtained when the method fails any artifacts, but this should not be interpreted as a good result). Magenta boxes with circles denote ARMBR, red boxes with squares denote MNE-SSP, green boxes with stars denote MNE Reg, cyan boxes with triangles denote ASR, and blue boxes with triangles denote EEGLAB Infomax ICA + ICLabel. Once again, ARMBR and Infomax ICA + ICLabel generally perform similarly to each other, and outperform the MNE methods, SSP and Reg. ASR exhibits the lowest RMSE among all tested methods. As previously noted, this low RMSE arises from two contributing factors: (1) when ASR successfully identifies and removes blink-related components, it effectively suppresses artifacts while preserving underlying neural activity; and (2) in instances where ASR fails to detect or attenuate blink artifacts, the output remains largely unchanged from the input, resulting in an artificially low RMSE despite inadequate artifact suppression. ARMBR and ASR exhibited more consistent values of RMAE across subjects and training conditions—even for small training signal segments (consistent with our observations from the previous analysis with semi-synthetic signals). ICA+ICLabel shows larger variance across participants in the more-frontal channels when the training data set is small; when trained and tested on the same 5 min segment, however, it exhibits the best performance of all.

### 5.6. ERP analysis

To evaluate whether the blink-suppressed EEG signals retain task-relevant responses, we removed blink artifacts from the Nencki-Symfonia EEG/ERP dataset (see section 2) using ARMBR with Fp1 and Fp2 as blink reference channels. Unlike the original authors [16], we included all epochs from all participants in our analysis (they manually identified bad data segments to remove from their analysis, before applying ICA with manual component selection to remove eye-movement, cardiac, and muscle artifacts).

**Figure 12.**
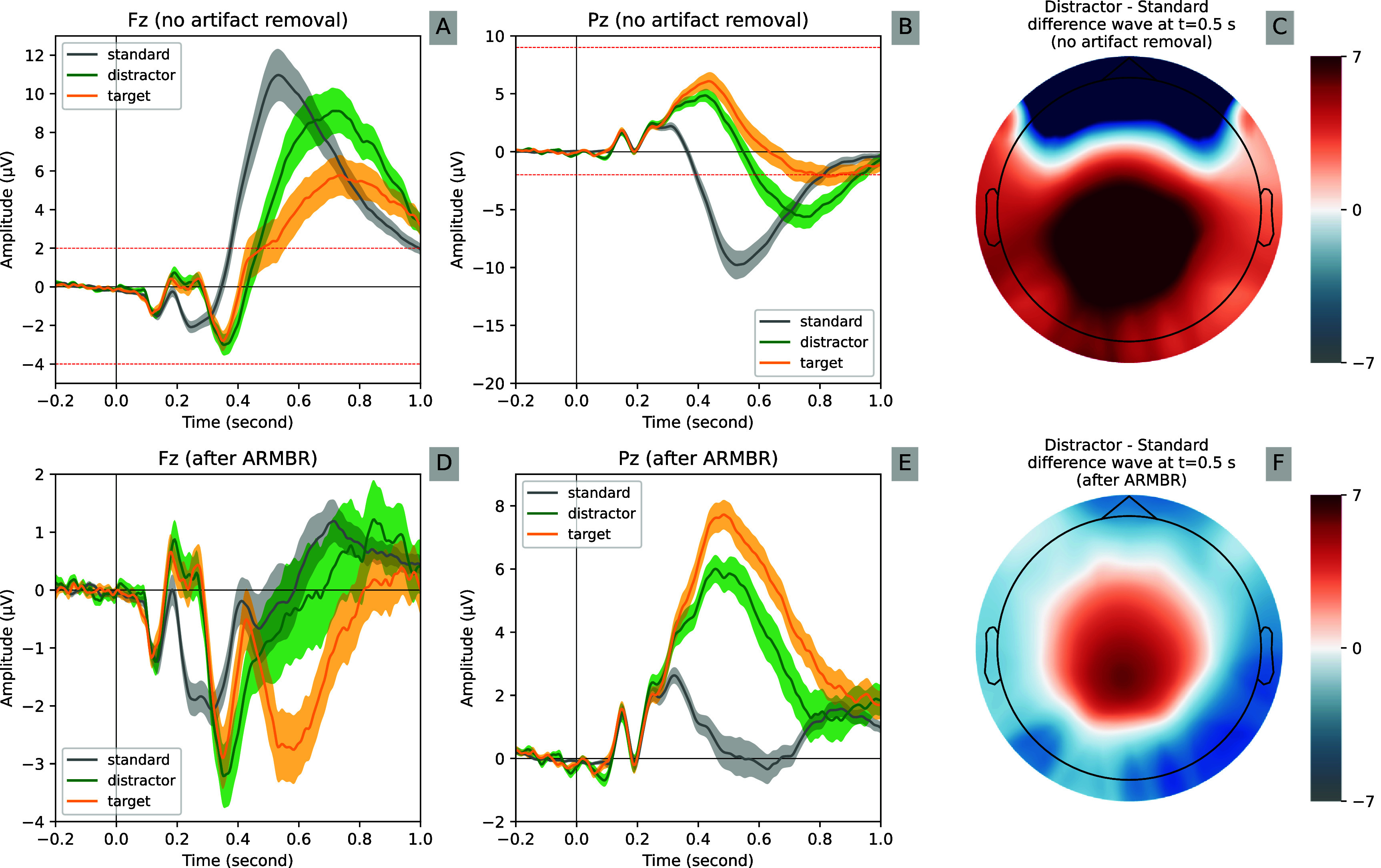
Event-related potential (ERP) analysis on the OpenNeuro *Nencki-Symfonia EEG/ERP dataset* before and after using ARMBR for blink removal during an oddball experiment where three stimuli were used: standard (in gray), distractor (in green), and target (in orange). Subplots (A) and (B) show the grand average ERP signal across all 42 participants for channels Fz and Pz, respectively, with no artifacts removed. Subplot (C) shows the corresponding scalp topography of the difference between the distractor and standard stimuli 500 ms after stimulus onset. Subplots (D), (E) and (F) show results of the same analysis after ARMBR has been used to remove blinks in the continuous data. The shaded colored regions represent the standard error across participants of the participants’ averaged waveforms. Note the difference in *y*-axis scaling: the red horizontal lines show where the *y*-axis limits of subplots (D) and (E) would fall in subplots (A) and (B), respectively. Subplots (D) and (E) look almost identical to the original authors’ analysis (figure 5 of Dzianok *et al* [16]).

Figure 12 shows the grand average ERP response to standard stimuli (gray traces), distractor stimuli (green traces), and target stimuli (orange traces), along with a scalp maps of the difference between responses to distractors and responses to standards, at 500 ms after presentation. The upper row of figure 12 shows the results from an analysis without artifact removal, illustrating the large influence of blinks. The lower row shows the results of applying ARMBR for blink artifact removal. The shaded region around each grand-average trace denotes the standard error across participants of the individual participants’ ERP averages. Subplots (A) and (D) of figure 12 show the average response from channel Fz. Subplots (B) and (E) of figure 12 show the average response from channel Pz. After blink artifact removal using ARMBR, the ERP signal becomes dominant in the topographical plot in subplot (F) of figure 12. The average ERP signals we show in subplots (D) and (E) of figure 12 are visually almost identical to those of figure 5 of Dzianok *et al* [16]. Hence, ARMBR successfully replicated the results of Dzianok *et al*’s more-complicated expert-dependent pipeline, preserving the desired ERP signals while removing the unwanted blink artifacts fully automatically.

## 6. Potential online application

**Figure 13.**
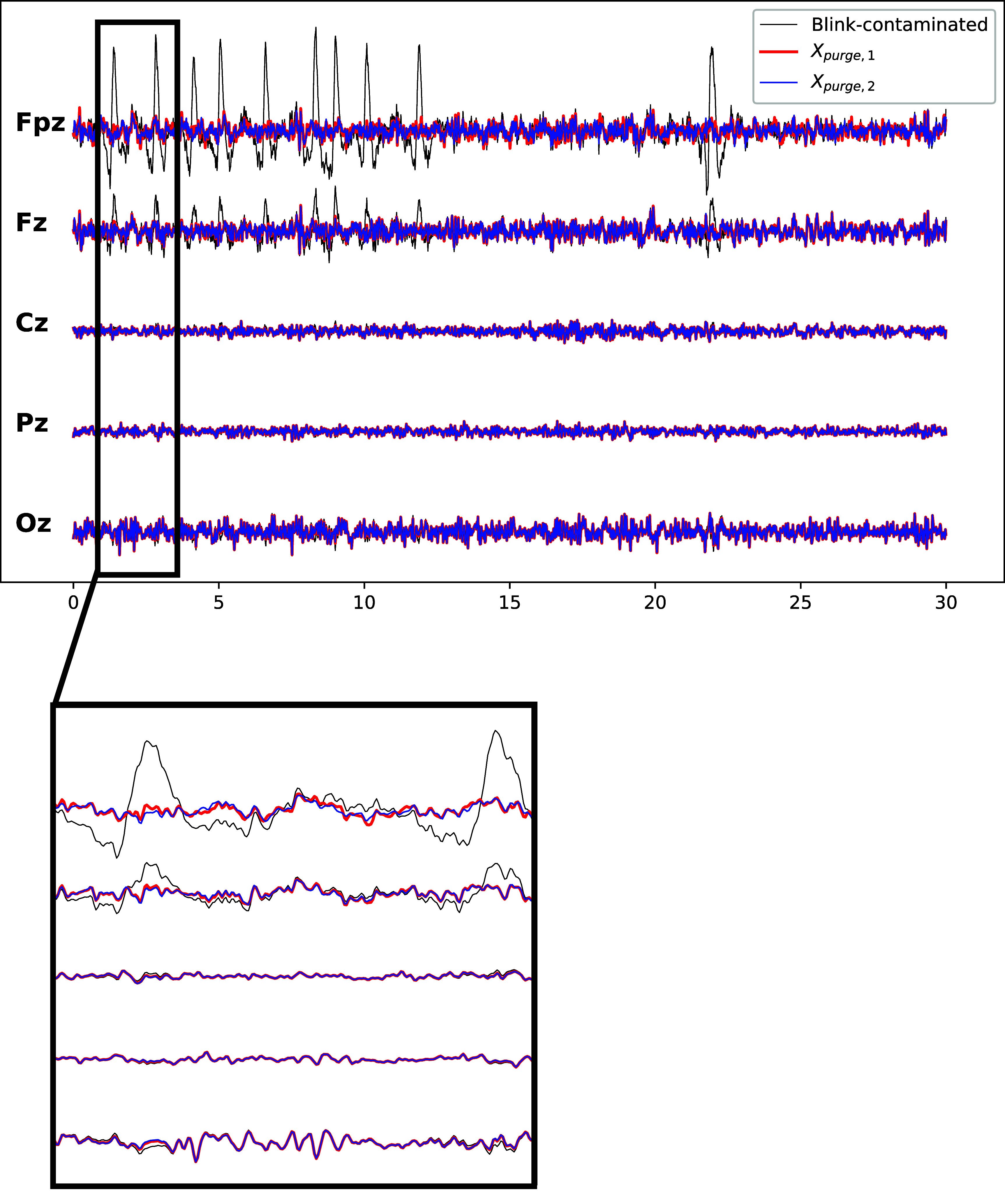
Online ability of ARMBR visualized on 30 s-long EEG recording from the real dataset participant 1 from channels Fp1, Fp2, Fz, Cz, Pz, and Oz. The black traces represent the blink-contaminated EEG signals. The red traces represent $X_\mathrm{purged,1}$ — ARMBR-cleaned test signals when we train on the first 5 min. The blue traces represent $X_\mathrm{purged,2}$ — ARMBR-cleaned test signals when we train on the second 5 min. The bottom subplot provides a better visual by zooming over the time range of 1–3 s.

The ability of ARMBR to be employed as an online blink-removal method was illustrated in figure 10. In the second to last row of figure 10, we trained on the first 5 min and test on the second 5 min. In the last row of figure 10, we train and test of the last 5 min. The performance of ARMBR (magenta circles) is similar in both cases. Here, we show in figure 13, 30 s of the EEG recording from the real dataset of participant 1 (testing EEG segment) from channels Fp1, Fp2, Fz, Cz, Pz, and Oz (we use this reduced set of channels for simplicity). The black traces in figure 13 represent the blink-contaminated EEG signals. The red traces represent ARMBR-cleaned test signals when we train on the first 5 min (denoted $X_\mathrm{purged,1}$), and the blue traces represent ARMBR-cleaned test signals when we train on the second 5 min (denoted $X_\mathrm{purged,2}$). The bottom subplot of figure 13 provides a better visual by zooming over the time range of 1 to 3 s. We can observe from figure 13 that (a) both $X_\mathrm{purged,1}$ and $X_\mathrm{purged,2}$ are free of major blink artifacts and (b) both $X_\mathrm{purged,1}$ and $X_\mathrm{purged,2}$ overlap significantly.

## 7. Discussion

We have reported on the performance of ARMBR, a novel linear subspace method for removing eye-blink (and potentially other) artifacts from multi-channel EEG. It relies on a reference signal, but unlike some competing methods, it does not need to interpret the reference signal as ground truth of the electrooculographic artifact. Some methods—including the MNE Regression method studied here [34] and others [22, 25]—make this assumption, and therefore typically require dedicated EOG channels. In post-hoc analyses, dedicated EOG channels may not be available; in their absence, a blink reference signal can be computed using channels close to the eye (for example, Fp1 and Fp2, which we used in the current study when using the MNE Reg method). However, this choice results in a suboptimal solution in which the EEG content of the channels in question is also targeted for removal. In this scenario, MNE Reg would generate a blink component using a linear combination of Fp1 and Fp2, and later set both channels to zero. After blink removal, the rank of the EEG data matrix is therefore reduced by the number of EEG channels *z* used to generate a blink reference signal (*z* = 2 when using Fp1 and Fp2). In contrast, ARMBR does not require a dedicated EOG electrode for measuring biological signals that can be assumed to reflect the ground truth. Rather, any heavily blink-affected channel or group of channels (though preferably as close to the eyes as possible, such as Fp1 and Fp2) could be used to extract a blink reference signal, which is then simplified to pulses so that the detailed EEG signal content is no longer targeted in the removal pipeline; in the output, the rank of the dataset is reduced by no more than 1.

In contrast, BSS algorithms such as ICA do not require an accurate, dedicated blink reference sensor. The concomitant disadvantage is that the user’s judgment is required in selecting blink components to be removed from the data. Therefore (at least, in the absence of auxiliary automation such as ICLabel), the user’s level of expertise is crucial to remove unwanted blink artifacts successfully. ARMBR aims to avoid this confound, automatically identifying blink components and thereby offering an objective solution to the problem. For this reason, we tested it only against other algorithms that fall within the same scope, i.e. algorithms that can be used ‘hands free’ without intervention or parameterization. The MNE SSP algorithm can be used in this way; for ICA, this also can be achieved by pairing the algorithm with auxiliary automation such as ICLabel.

Developed with the same goal of reducing reliance on user expertise and intervention, ICLabel provides a potential solution to this problem by automatically flagging blink components (among other components, such as heart beats and muscle artifacts). The use-cases for ICA+ICLabel are somewhat distinct from the use-cases for ARMBR: ICA requires a relatively large amount of data and so is best suited for offline analyses, but it has been developed to identify additional artifacts such as pulse, muscle and electrode movement; by contrast, ARMBR can use much smaller training data sets and is therefore potentially a more agile component of online neurotechnology systems, but it specifically targets only one artifact species at a time, and so far we have only optimized and assessed its ability to detect blinks. Despite this mismatch, we included ICA+ICLabel in this analysis because we found that it generally performed well, outperforming the other non-ARMBR methods, *despite* being tested outside of its usual scope, on the small datasets characteristic of the online use-case. As expected, it did suffer somewhat in this context, only equaling ARMBR’s performance on the small datasets when the channel count was kept low, and being rather more sensitive to variation in the amount of blinking in the training data. In short, ICA performed as well as ARMBR when conditions were ideal (large enough amount of data; low enough noise; few enough blinks; experienced enough user—or successful enough application of ICLabel to substitute for expertise) but ARMBR was more robust to less-than-ideal conditions. Within the context of a narrowly-defined problem such as blink artifact removal, this is not surprising, since ARMBR directly targets the particular signal or artifact (in this case, fitting a linear subspace that directly explains the difference between signal segments with and without high blink-like excursions). While this makes any one instantiation of ARMBR more specialized and far less flexible than ICA, the flip-side is that it attacks the problem more directly rather than relying on the data quantity and SNR being high enough to allow a particular artifact to be found indirectly via an analysis that optimizes a different criterion (namely, statistical independence).

Recently, several approaches based on deep learning algorithms have also been applied to the EEG denoising problem [12, 49, 51, 52] and present interesting features, such as the possibility of removing both ocular and muscle artifacts simultaneously. Even though such methods perform very well in some situations, they present various limitations that are worth mentioning. For instance, despite some complex neural networks being trained on thousands of EEG data segments, larger amounts of data for training and testing remain necessary for the model to generalize to all possible artifacts [52]. Moreover, the majority of these methods were trained on a single EEG channel, which means that they fail to take advantage of the linear nature of volume conduction to extract artifacts that occupy a linear subspace of multi-dimensional mixed signals. Lastly, most of the methods used supervised learning which requires large amounts of expert-annotated data—a feature that once again makes them heavily dependent on individual expertise [49]. In addition to the need for single-artifact transparency and zero user expertise, we also excluded these algorithms for the practical reason of restricting our scope to algorithms that the reader could readily download and use ‘out of the box’; at the time of writing, we found no such implementation of the deep-learning algorithms.

A key feature of algorithms for extracting signals from noisy high-dimensional signals is regularization, i.e. the penalization of unnecessary complexity in the solution. This has been widely used in supervised EEG classification algorithms, as well as pattern and filter extraction from EEG data. An example is in the estimation of temporal response functions, a method generally used to discover a mapping between some feature(s) of a complex sensory stimulus and the neural response using a regularized linear regression [11]. In [11], regularization is used to overcome the ill-posed nature of the estimation problem and prevent over-fitting. AMBR achieves the encouraging results reported in this paper without incorporating regularization explicitly—however it is incorporated implicitly by simplifying the targeted reference signal. By simplifying each blink to a binary pulse (most of whose variance would be explained by EOG and very little by EEG or noise), ARMBR prevents rich EEG content and noise from influencing the regression solution. In terms of the linear algebra of multiple regression, the sparse nature of the regression target (pulses) means that its covariance matrix is highly diagonal, and it becomes redundant to add a diagonal ridge to it (which is how one would implement L2 regression, the most common type of regression). In this way, the simplification and sparsification of the regression target serves as an intrinsic way of limiting the undesired removal of brain waves via overly complex solutions.

Algorithms exhibiting ARMBR’s robustness and low computational intensiveness are of particular use in brain–computer interfacing (BCI) applications, which have gained high popularity in the neuro-scientific community [48] in the last two to three decades. In certain BCI experiments, ocular and blink data can serve as important features [53]. In others, real-time blink artifact removal becomes necessary, especially in EEG-based real-time applications that involve the extraction of brain waves from electrodes mostly affected by blinks. For example, the study in [38] investigated the feasibility of a passive BCI that uses EEG to monitor changes in mental state on a single-trial basis. There, the importance of the central and frontal (the latter in particular being heavily influenced by blink artifacts) was highlighted in a fatigue detection application [38]. In [38], the blink artifacts were removed offline using the ADJUST ICA method [37]. For a real-time implementation of the method in [38], real-time blink-removal methods like ARMBR are desirable, since ARMBR could be implemented using participant-specific blink spatial filters generated quickly and inexpensively from prior training data segments. Our focus on algorithms suitable for online applications might be seen as an argument for excluding ICA, which is well known to suffer when using small amounts of training data, especially when the channel count is larger. Its sensitivity to training set size and channel count were indeed evident in our results, but it nonetheless performed better than the two MNE methods under most conditions, performed as well as ARMBR when the channel count was low, and even exceeded ARMBR’s performance in preserving higher-frequency content when the amount of training data was sufficiently large. For this reason, we found it instructive to include its results in the report.

We envision ARMBR being usable both by first-time EEG researchers and by experts in the field. We provide a Python and a MATLAB package of ARMBR with a generic code that can be easily implemented using MNE Raw objects and EEGLAB structures. Thus, ARMBR provides a user-friendly manner to generate reproducible results regardless of the level of expertise and experience of the user.

Finally, we should note that the same approach of backward regression against a reference signal can in principle be applied to any other form of artifact for which such a reference can be generated. One example might be reference signals that target saccadic eye movements (whose simplified forms are more akin to step functions than pulses): these might be detected by combining and thresholding the numerical *derivatives* of the frontal channels’ EEG signals, rather than the raw signal. It is even possible to remove power-line artifacts in this way, by modeling a sine-wave of unknown phase as the sum of a cosine and sine reference signal—the reference signal B in (3)–(5) would then be two-dimensional: one column of B would contain $\cos(t)$ and the other $\sin(t)$, and a spatial filter is estimated for, and used to remove, the spatial projection of each of the two components. (While this example illustrates the flexibility of the algorithm in principle, and while we have found it to work very well in some cases, we do not suggest that it is worth reducing the rank of the EEG signal by 2 purely for the purpose of removing an artifact that might better be removed by a low-pass filter or a notch filter centered on 50–60 Hz.)

## 8. Summary/conclusion

In this paper, we presented an automatic method of artifact removal from EEG signals, denoted ARMBR. This method takes a simple multivariate regression approach to computed a spatial weighting across channels, which can then be used as a spatial filter to estimate an artifact and project it out of the linear signal space. The spatial filter is optimized by regressing against a highly simplified reference signal—this has the effect of regularizing the estimation and hence avoiding over-subtraction of meaningful EEG components, without requiring dedicated channels that specifically aim to measure the artifact. It also allows effective learning from very small data-sets, making the algorithm highly suitable for online neurotechnology applications.

In the example application presented in this article—specific removal of eye-blink artifacts—the method has only one hyperparameter: an amplitude threshold for roughly discriminating blink from non-blink signal segments. We incorporate a cross-validation method for optimizing this hyperparameter, hence making the method entirely ‘hands-free’—i.e. removing the need for expert judgment.

We have reported ARMBR’s performance in comparison with other readily-downloadable hands-free implementations, with an emphasis on algorithms also do not demand large training datasets—the exception, an algorithm based on ICA, was included because it performs competitively *despite* running on a smaller data-set than it is designed to use. The algorithms were SSP from the MNE toolbox, forward regression from the MNE toolbox, and Infomax ICA coupled with ICLabel, from the EEGLAB toolbox.

In our first test, we constructed 10 small ‘semi-synthetic’ EEG recordings by linearly combining clean and blink artifact segments extracted from real EEG signals. Comparison between clean and blink-suppressed signals showed that the root-mean-squared error of the ARMBR method was smaller, while the SNR and Pearson correlation were greater, compared to all other methods. These results were statistically significant with *p* < 0.05 in two-sided Wilcoxon signed-rank tests, and ANOVA.

We then used real EEG datasets in which the ground truth was unknown. The first was from 16 human participants performing a natural-speech listening task. We computed semi-normalized correlation coefficients *R*, to assess the amount of change each algorithm wrought in blink-affected epochs (where we expect large changes) as well as unaffected epochs (where we would hope to see no change) and less-affected channels (where we would hope for little or no change). We also used a relative mean-absolute-error criterion to quantify the effect on PSD in EEG bands of interest (where we would also hope to see little change). By these criteria, we found that ARMBR exhibited consistently higher performance than the MNE SSP and MNE Reg methods, and comparable performance to the ICA + ICLabel method—with a moderate advantage for ARMBR when training signals had short length and/or high channel counts as might be expected; ARMBR also exhibited somewhat greater robustness to between-subject variations in blink rate.

The second real EEG dataset came from 42 human participants performing a visual oddball task. We showed that ARMBR successfully preserved the expected ERP signals by removing the unwanted blink artifacts fully automatically, with the results almost identically matching the dataset owners’ previous analysis using a hand-guided ICA-based artifact removal method.

We conclude that ARMBR provides a powerful approach to EEG artifact removal that can be used without expert guidance and without demanding large training datasets. Though we have only thoroughly demonstrated its use in the context of blink artifact removal, its general approach can readily be adapted to target other artifact types—a possibility that invites further study.

## 9. Limitations

• ICA relies on statistical properties that require sufficiently long data segments to perform optimally. Our semi-synthetic analysis, based on 15 s segments, does not reflect ideal conditions for ICA, which is better suited for longer recordings. In contrast, ARMBR is event-driven and less dependent on data length. Method comparisons are sensitive to parameter choices. Suboptimal settings can lead to misleading conclusions. For example, the performance of ICA+ICLabel on some semi-synthetic participants in figure 8 could be improved by simply lowering the classification threshold (e.g. from 0.9 to 0.8), even on short segments. However we have confined the scope of the current study to use all implementations of the respective methods with out-of-the-box settings, assuming that the user will not necessarily have the expertise necessary to adjust parameters.
• We benchmarked ARMBR against ASR [26]. On the real data, as illustrated in figures 10 and 11, ASR exhibited high correlation coefficients (*R*) and low RMSE values even in blink-contaminated epochs and channels. We believe these results could potentially stem from failure to detect blinks. Similar to the case with ICA, this may be because we used out-of-the-box parameter values without user intervention. With greater expertise and intervention, users may improve ASR results either by manually selecting clean EEG segments as training data or by adjusting the the way this training is automatically bootstrapped by changing the artifact-detection threshold parameter [6].

We acknowledge the above-mentioned factors and present results with this context in mind.

## Figures and Tables

**Figure A1.**
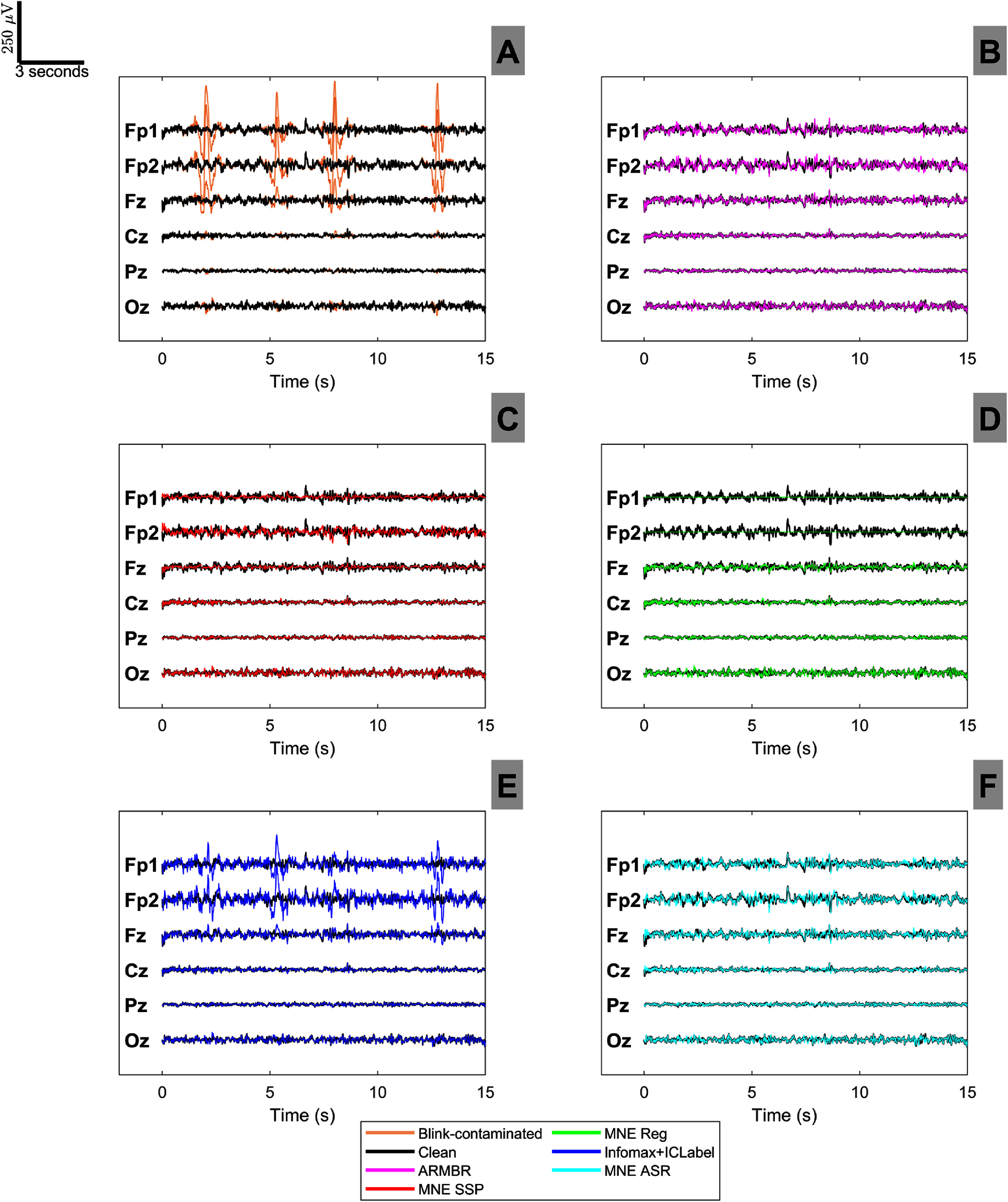
Methods comparison graphs for participant 1 of the semi-synthetic dataset.

**Figure A2.**
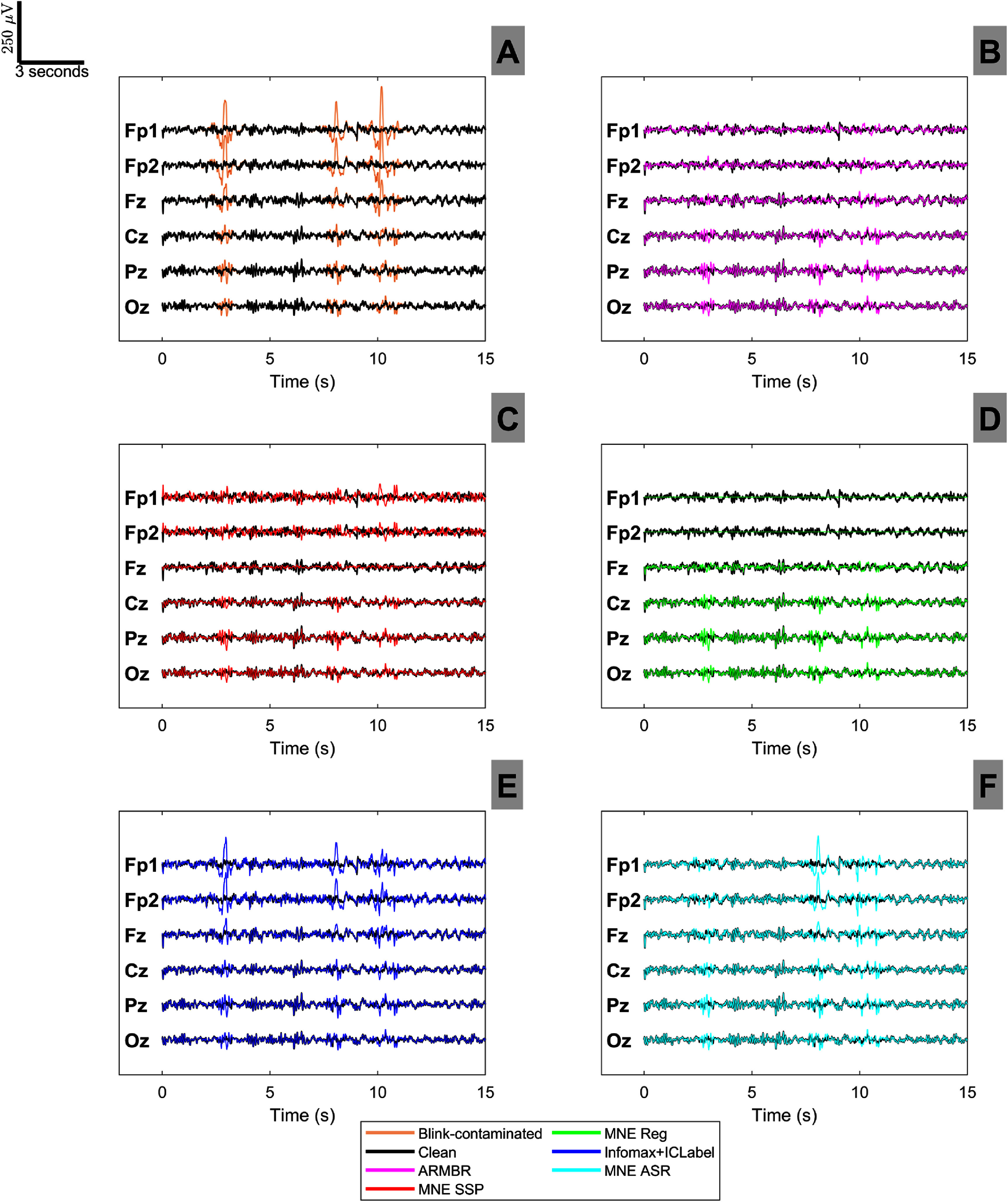
Methods comparison graphs for participant 2 of the semi-synthetic dataset.

**Figure A3.**
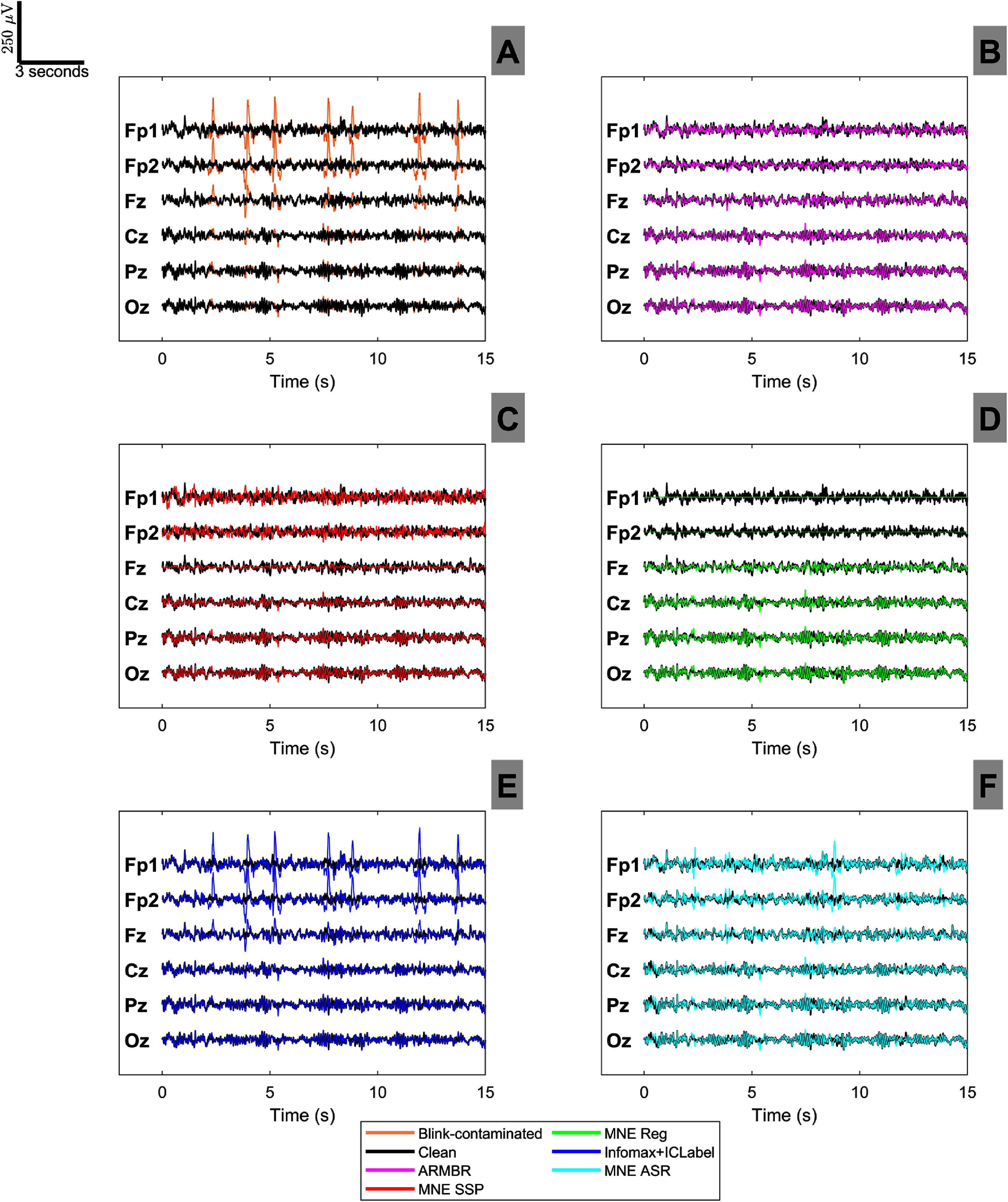
Methods comparison graphs for participant 3 of the semi-synthetic dataset.

**Figure A4.**
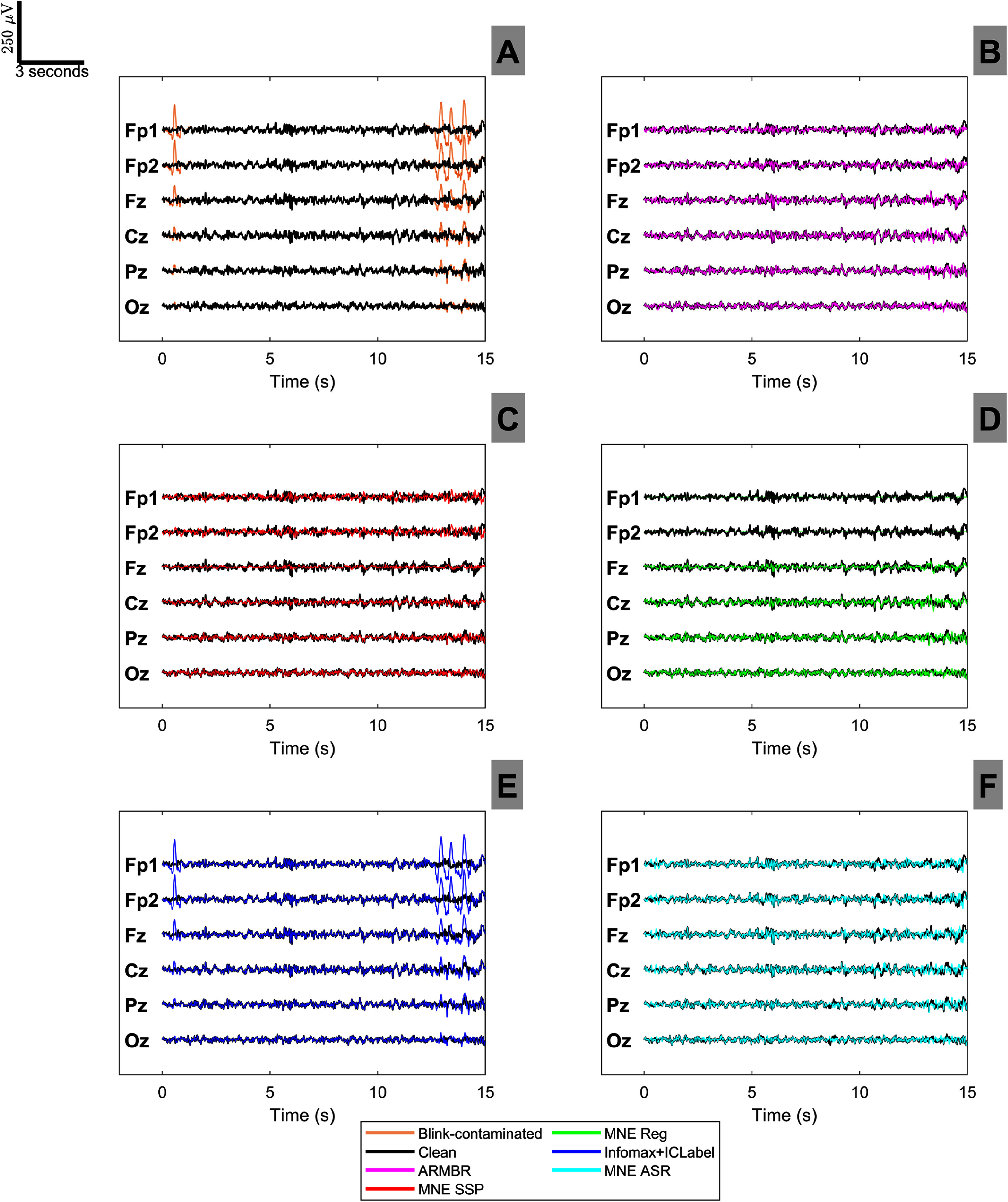
Methods comparison graphs for participant 6 of the semi-synthetic dataset.

**Figure A5.**
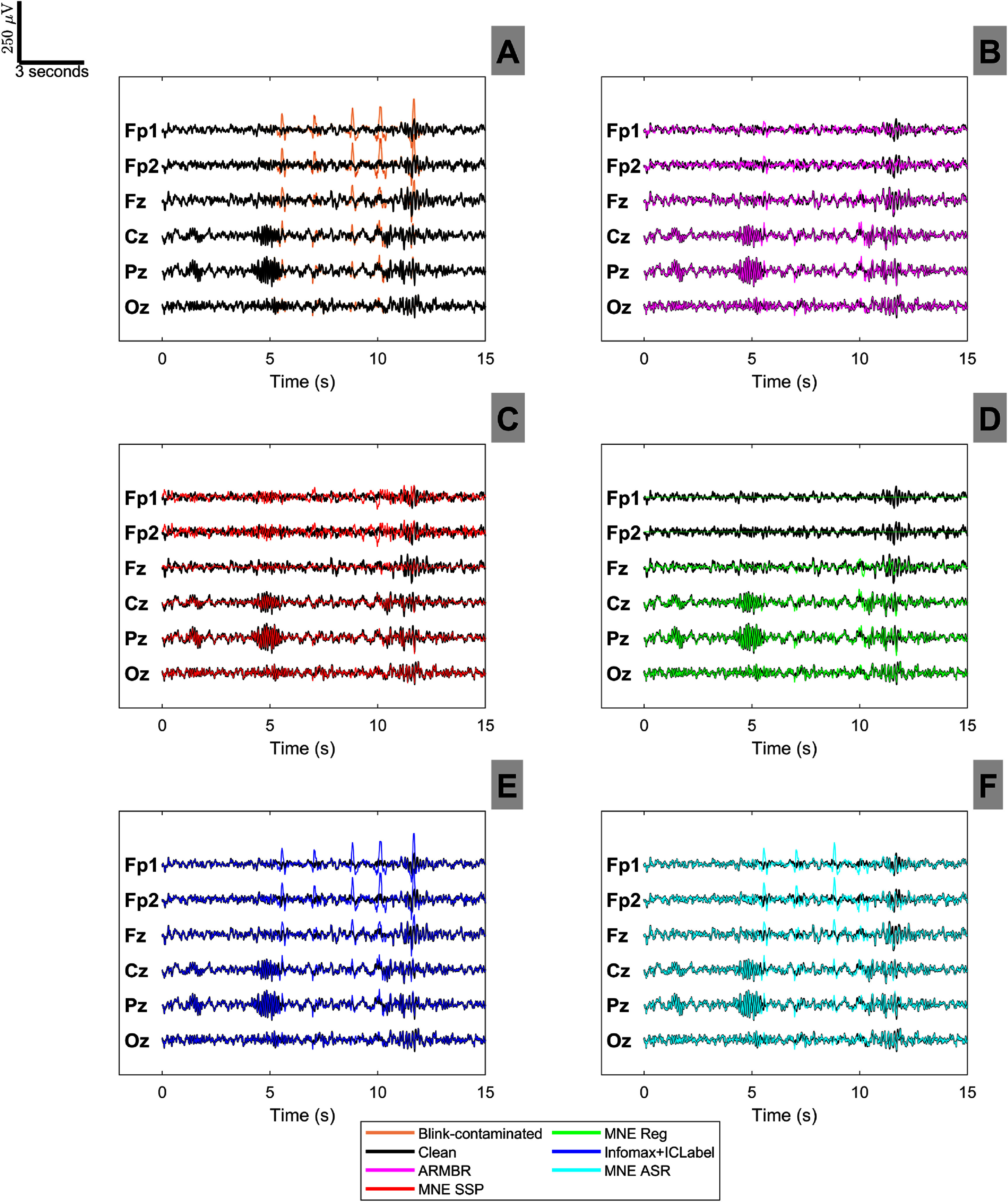
Methods comparison graphs for participant 8 of the semi-synthetic dataset.

**Figure A6.**
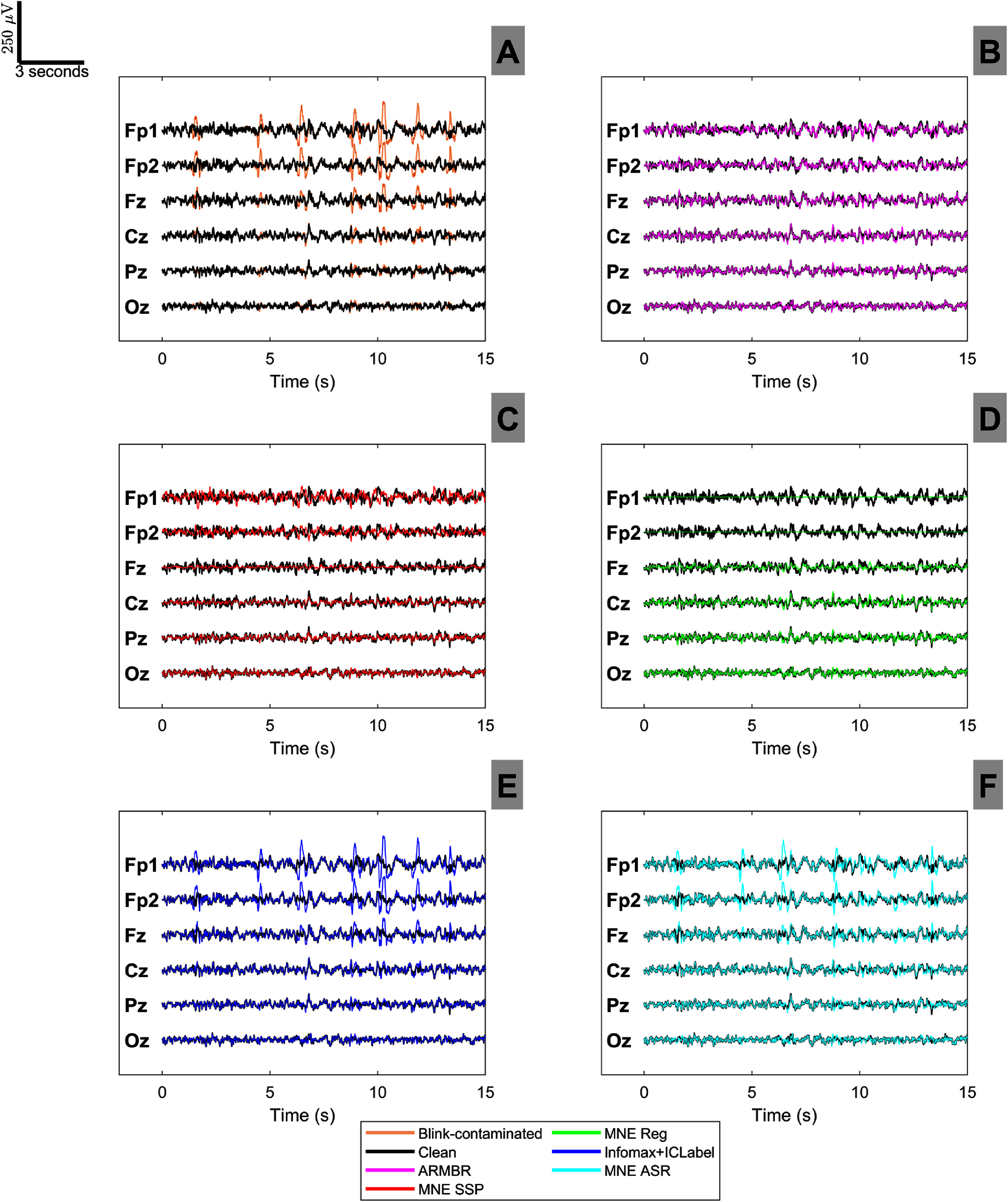
Methods comparison graphs for participant 11 of the semi-synthetic dataset.

**Figure A7.**
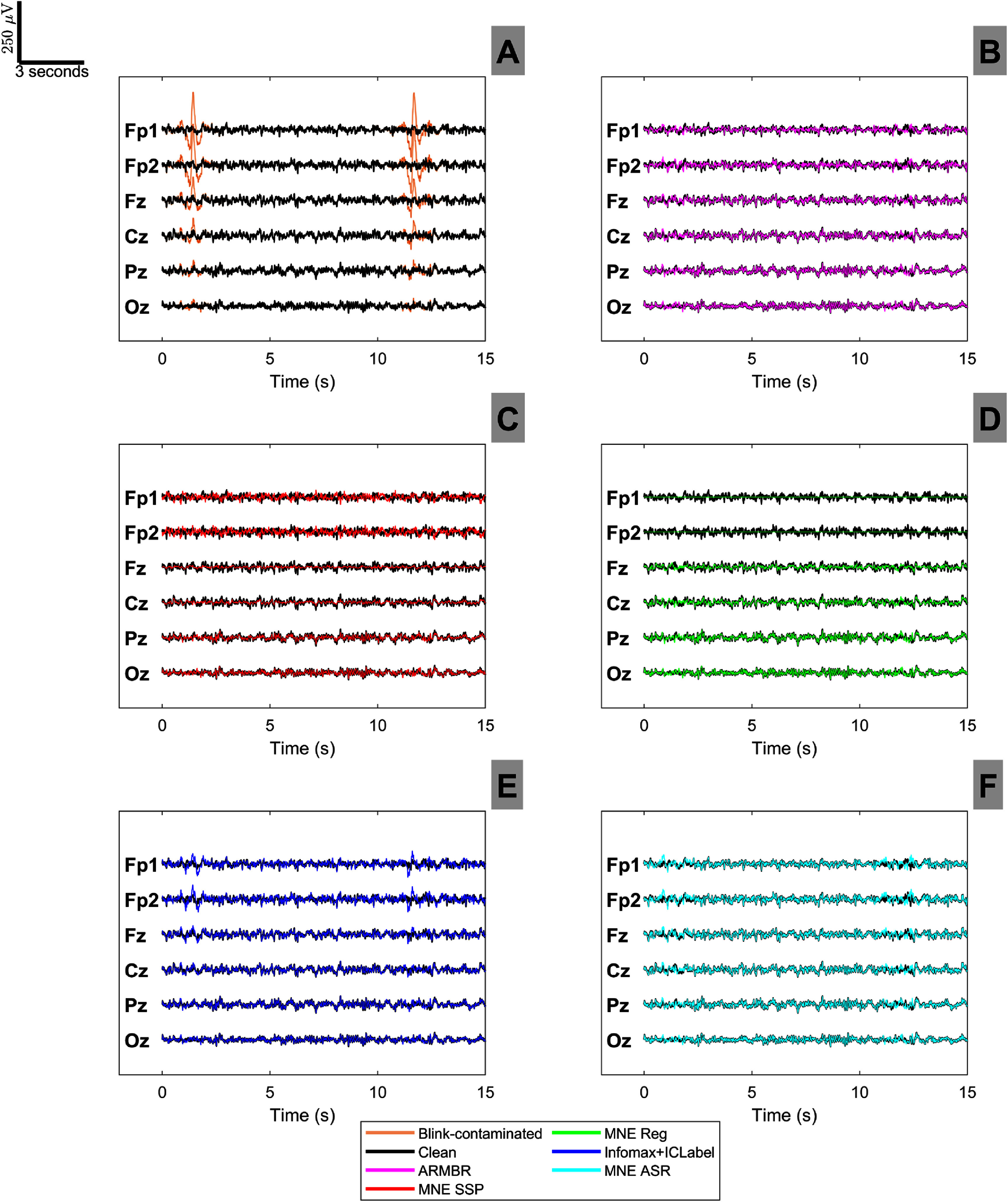
Methods comparison graphs for participant 13 of the semi-synthetic dataset.

**Figure A8.**
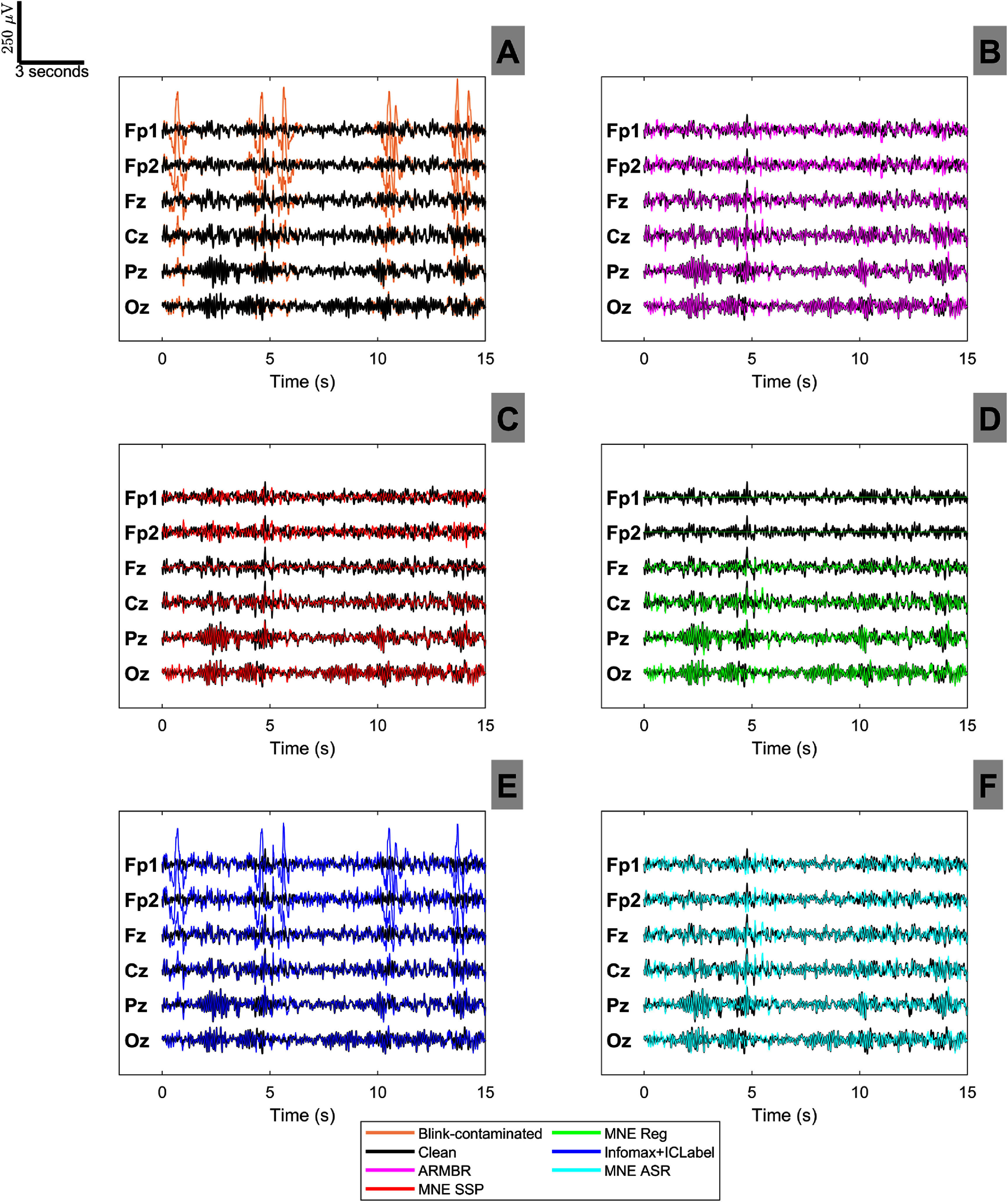
Methods comparison graphs for participant 14 of the semi-synthetic dataset.

**Figure A9.**
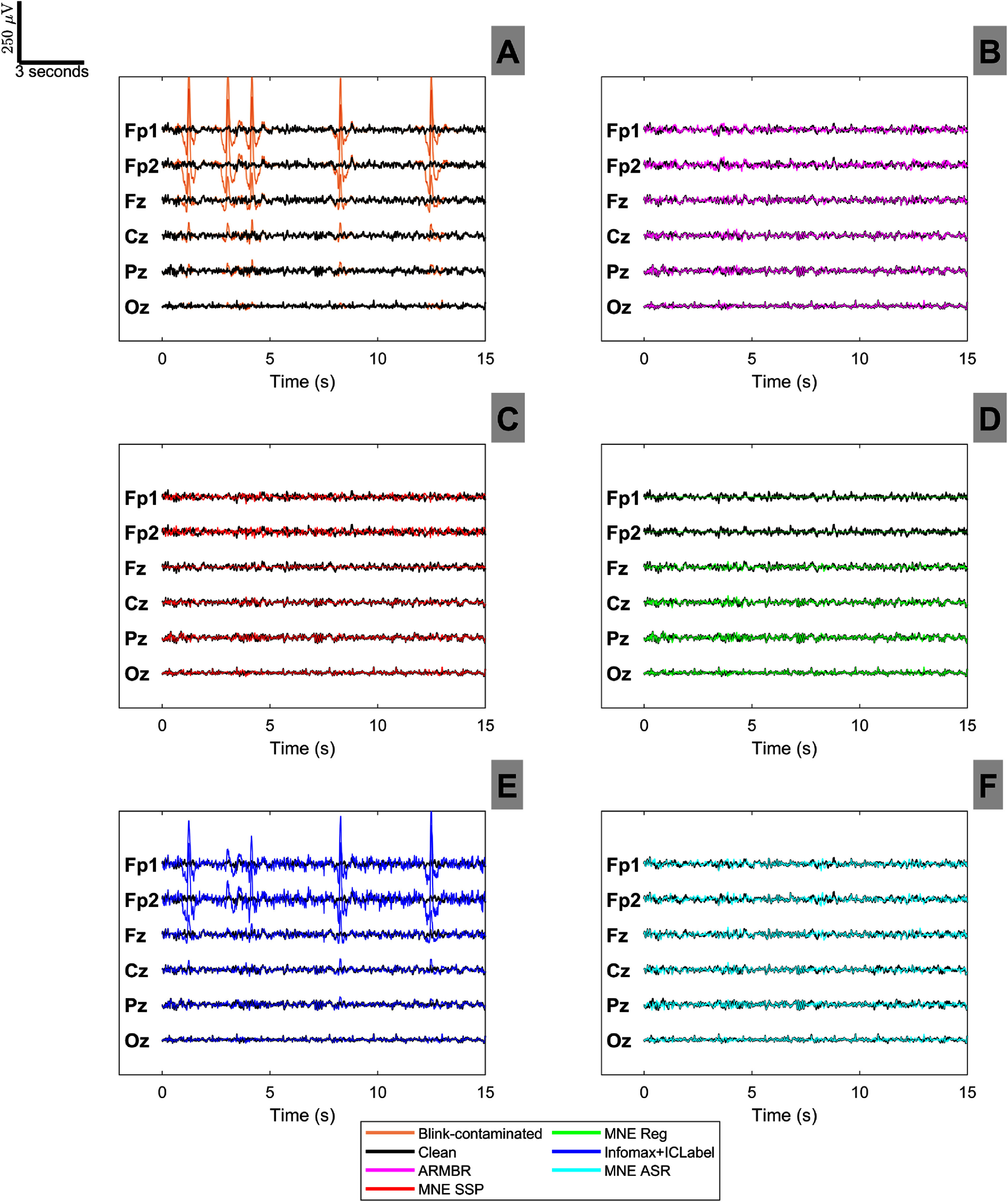
Methods comparison graphs for participant 17 of the semi-synthetic dataset.

**Figure A10.**
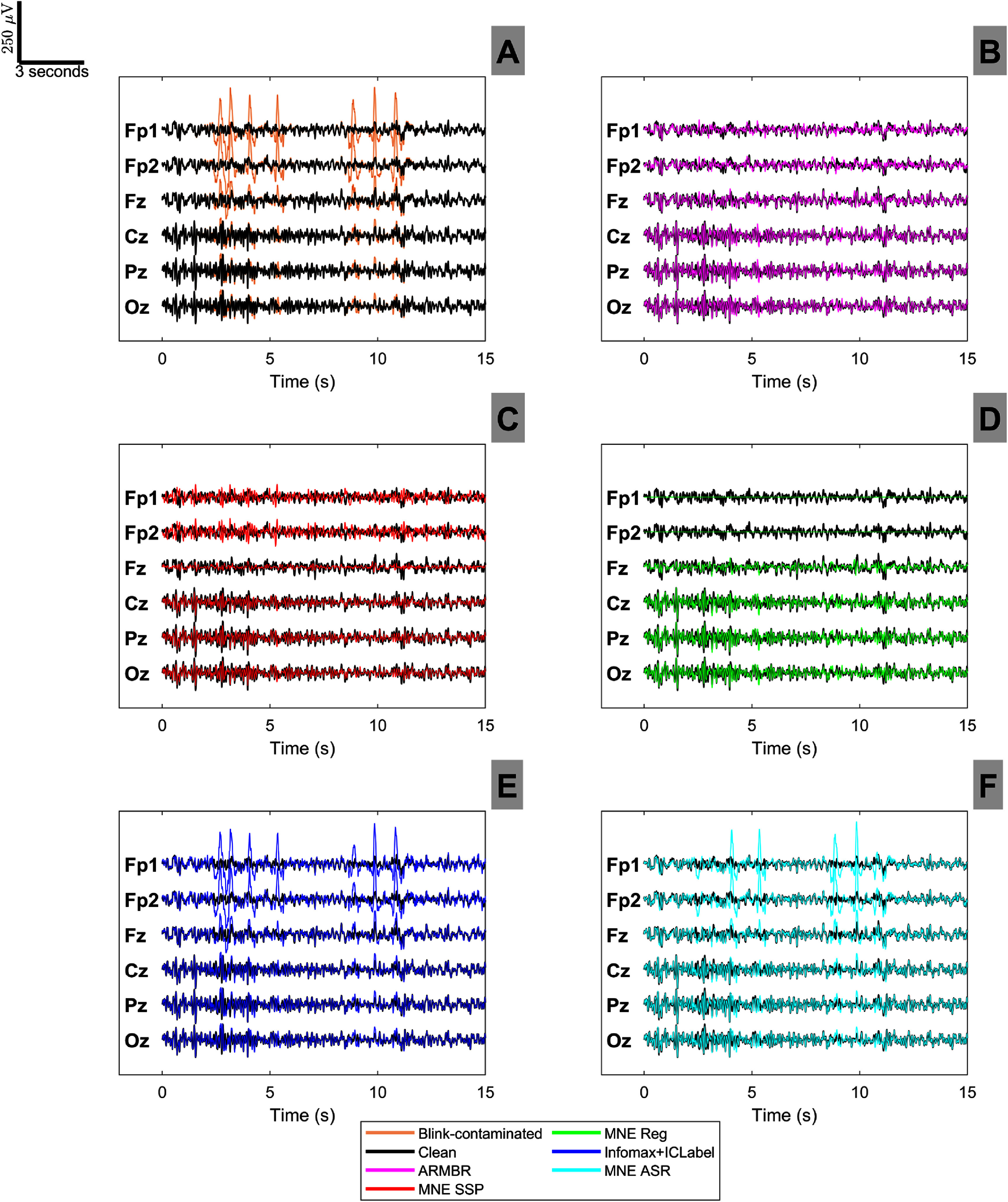
Methods comparison graphs for participant 18 of the semi-synthetic dataset.

## Data Availability

The data that support the findings of this study are openly available at the following URL/DOI: https://datadryad.org/stash/dataset/doi:10.5061/dryad.070jc.

## Appendix Methods comparison graphs

Tables A1–A3 show the RMSE, SNR, and $\rho_\mathrm{pearson}$ for our proposed method as well as the 4 alternative methods of for (a) all channels, (b) the channels most affected by blinks, and (c) the channels least affected by blinks, respectively, for each semi-synthetic dataset participant. Moreover, figure A1 through figure A10 visually present the results of all blink removal methods discussed in the paper applied to the different participants’ EEG signals. These methods are ARMBR (magenta), MNE SSP (red), MNE regression (green), ASR (cyan), and EEGLAB ICA Infomax + ICLabel (blue traces). The black traces correspond to the clean EEG signal (ground truth). The orange traces are the synthetically blink-contaminated signals. For simplicity, a subset of EEG channels is used for this demonstration (Fp1, Fp2, Fz, Cz, Pz, and Oz).

**Table A1.** RMSE, SNR, and Pearson correlation of each participant for all tested methods from all channels.

Participant	MNE SSP	MNE Reg	EEGLAB ICA + ICLabel	ASR	ARMBR
1	RMSE = 3.08 SNR = 3.05 *ρ* = 0.8	RMSE = 2.93 SNR = 3.05 *ρ* = 0.83	RMSE = 3.82 SNR = 2.42 *ρ* = 0.84	RMSE = 2.52 SNR = 3.52 *ρ* = 0.9	RMSE = 2.49 SNR = 3.7 *ρ* = 0.91
2	RMSE = 5.01 SNR = 1.16 *ρ* = 0.63	RMSE = 4.47 SNR = 1.5 *ρ* = 0.75	RMSE = 4.92 SNR = 1.2 *ρ* = 0.8	RMSE = 4.42 SNR = 1.59 *ρ* = 0.82	RMSE = 4.2 SNR = 1.76 *ρ* = 0.8
3	RMSE = 4.51 SNR = 2.97 *ρ* = 0.76	RMSE = 3.47 SNR = 3.78 *ρ* = 0.87	RMSE = 5.45 SNR = 2.2 *ρ* = 0.82	RMSE = 4.13 SNR = 2.99 *ρ* = 0.87	RMSE = 3.04 SNR = 4.28 *ρ* = 0.92
6	RMSE = 4.24 SNR = 2.31 *ρ* = 0.7	RMSE = 3.36 SNR = 3.13 *ρ* = 0.82	RMSE = 4.68 SNR = 2.13 *ρ* = 0.82	RMSE = 3.65 SNR = 2.64 *ρ* = 0.83	RMSE = 2.39 SNR = 4.51 *ρ* = 0.92
8	RMSE = 5.48 SNR = 2.54 *ρ* = 0.72	RMSE = 3.97 SNR = 3.71 *ρ* = 0.86	RMSE = 4.2 SNR = 3.61 *ρ* = 0.9	RMSE = 3.56 SNR = 4.2 *ρ* = 0.91	RMSE = 3.11 SNR = 4.67 *ρ* = 0.93
11	RMSE = 4.99 SNR = 2.11 *ρ* = 0.68	RMSE = 3.96 SNR = 2.89 *ρ* = 0.82	RMSE = 4.59 SNR = 2.56 *ρ* = 0.86	RMSE = 3.75 SNR = 3.22 *ρ* = 0.89	RMSE = 2.83 SNR = 4.16 *ρ* = 0.92
13	RMSE = 3.95 SNR = 2.45 *ρ* = 0.71	RMSE = 2.93 SNR = 3.53 *ρ* = 0.85	RMSE = 2.54 SNR = 4.22 *ρ* = 0.92	RMSE = 2.14 SNR = 4.74 *ρ* = 0.94	RMSE = 2.14 SNR = 4.77 *ρ* = 0.93
14	RMSE = 6.37 SNR = 2.73 *ρ* = 0.7	RMSE = 5.92 SNR = 2.73 *ρ* = 0.8	RMSE = 8.99 SNR = 1.39 *ρ* = 0.78	RMSE = 5.24 SNR = 3.17 *ρ* = 0.87	RMSE = 4.71 SNR = 3.7 *ρ* = 0.89
17	RMSE = 2.69 SNR = 2.93 *ρ* = 0.77	RMSE = 2.28 SNR = 3.37 *ρ* = 0.86	RMSE = 5.69 SNR = 0.34 *ρ* = 0.71	RMSE = 2.83 SNR = 2.3 *ρ* = 0.81	RMSE = 2.18 SNR = 3.51 *ρ* = 0.89
18	RMSE = 6.45 SNR = 2.24 *ρ* = 0.66	RMSE = 5.06 SNR = 3 *ρ* = 0.83	RMSE = 6.95 SNR = 2.04 *ρ* = 0.84	RMSE = 5.91 SNR = 2.65 *ρ* = 0.85	RMSE = 3.69 SNR = 4.27 *ρ* = 0.92

**Table A2.** RMSE, SNR, and Pearson correlation of each participant for all tested methods from blink-affected channels.

Participant	MNE SSP	MNE Reg	EEGLAB ICA + ICLabel	ASR	ARMBR
1	RMSE = 8.4 SNR = −0.57 *ρ* = −0.03	RMSE = 6.46 SNR = 0.54 *ρ* = 0.35	RMSE = 11.96 SNR = −2.12 *ρ* = 0.53	RMSE = 4.87 SNR = 1.76 *ρ* = 0.79	RMSE = 5.2 SNR = 1.49 *ρ* = 0.77
2	RMSE = 9.08 SNR = −1.42 *ρ* = −0.08	RMSE = 5.36 SNR = 0.71 *ρ* = 0.49	RMSE = 8.33 SNR = −1.09 *ρ* = 0.65	RMSE = 6.43 SNR = −0.02 *ρ* = 0.71	RMSE = 4.67 SNR = 1.31 *ρ* = 0.7
3	RMSE = 10.73 SNR = −1.41 *ρ* = −0.11	RMSE = 5.98 SNR = 1.14 *ρ* = 0.51	RMSE = 13.23 SNR = −2.32 *ρ* = 0.5	RMSE = 6.95 SNR = 0.46 *ρ* = 0.7	RMSE = 4.47 SNR = 2.38 *ρ* = 0.81
6	RMSE = 9.1 SNR = −1.13 *ρ* = −0.12	RMSE = 5.98 SNR = 0.67 *ρ* = 0.4	RMSE = 11.55 SNR = −2.15 *ρ* = 0.51	RMSE = 4.82 SNR = 1.64 *ρ* = 0.74	RMSE = 4.05 SNR = 2.41 *ρ* = 0.8
8	RMSE = 11.47 SNR = −1.54 *ρ* = −0.05	RMSE = 6.59 SNR = 0.85 *ρ* = 0.49	RMSE = 9.17 SNR = −0.56 *ρ* = 0.69	RMSE = 6.84 SNR = 0.72 *ρ* = 0.73	RMSE = 5.14 SNR = 1.94 *ρ* = 0.76
11	RMSE = 11.24 SNR = −1.05 *ρ* = −0.09	RMSE = 7.67 SNR = 0.61 *ρ* = 0.45	RMSE = 11.53 SNR = −1.13 *ρ* = 0.62	RMSE = 8.67 SNR = 0.1 *ρ* = 0.7	RMSE = 4.74 SNR = 2.72 *ρ* = 0.84
13	RMSE = 8.95 SNR = −1.41 *ρ* = −0.17	RMSE = 5.49 SNR = 0.7 *ρ* = 0.41	RMSE = 5.55 SNR = 0.66 *ρ* = 0.74	RMSE = 3.52 SNR = 2.64 *ρ* = 0.86	RMSE = 3.93 SNR = 2.16 *ρ* = 0.79
14	RMSE = 15.48 SNR = −1.96 *ρ* = −0.46	RMSE = 9.09 SNR = 0.32 *ρ* = 0.35	RMSE = 23.45 SNR = −3.77 *ρ* = 0.4	RMSE = 6.65 SNR = 1.67 *ρ* = 0.78	RMSE = 7.8 SNR = 0.99 *ρ* = 0.69
17	RMSE = 6.83 SNR = −1.12 *ρ* = −0.08	RMSE = 4.35 SNR = 0.79 *ρ* = 0.52	RMSE = 17.97 SNR = −5.31 *ρ* = 0.31	RMSE = 4.08 SNR = 1.05 *ρ* = 0.69	RMSE = 4.1 SNR = 1.06 *ρ* = 0.71
18	RMSE = 13.98 SNR = −1.81 *ρ* = −0.29	RMSE = 7.95 SNR = 0.63 *ρ* = 0.53	RMSE = 16.64 SNR = −2.56 *ρ* = 0.57	RMSE = 13.84 SNR = −1.76 *ρ* = 0.57	RMSE = 5.16 SNR = 2.5 *ρ* = 0.83

**Table A3.** RMSE, SNR, and Pearson correlation of each participant for all tested methods from blink less-affected channels.

Participant	MNE SSP	MNE Reg	EEGLAB ICA + ICLabel	ASR	ARMBR
1	RMSE = 2.48 SNR = 3.46 *ρ* = 0.89	RMSE = 2.53 SNR = 3.33 *ρ* = 0.88	RMSE = 2.9 SNR = 2.93 *ρ* = 0.88	RMSE = 2.26 SNR = 3.71 *ρ* = 0.91	RMSE = 2.18 SNR = 3.95 *ρ* = 0.92
2	RMSE = 4.55 SNR = 1.45 *ρ* = 0.71	RMSE = 4.37 SNR = 1.59 *ρ* = 0.77	RMSE = 4.53 SNR = 1.46 *ρ* = 0.81	RMSE = 4.19 SNR = 1.77 *ρ* = 0.83	RMSE = 4.15 SNR = 1.81 *ρ* = 0.81
3	RMSE = 3.81 SNR = 3.47 *ρ* = 0.86	RMSE = 3.18 SNR = 4.08 *ρ* = 0.91	RMSE = 4.57 SNR = 2.71 *ρ* = 0.86	RMSE = 3.81 SNR = 3.27 *ρ* = 0.89	RMSE = 2.88 SNR = 4.5 *ρ* = 0.93
6	RMSE = 3.69 SNR = 2.7 *ρ* = 0.8	RMSE = 3.07 SNR = 3.41 *ρ* = 0.87	RMSE = 3.91 SNR = 2.61 *ρ* = 0.86	RMSE = 3.51 SNR = 2.75 *ρ* = 0.84	RMSE = 2.21 SNR = 4.75 *ρ* = 0.94
8	RMSE = 4.8 SNR = 3 *ρ* = 0.8	RMSE = 3.67 SNR = 4.03 *ρ* = 0.9	RMSE = 3.64 SNR = 4.08 *ρ* = 0.92	RMSE = 3.19 SNR = 4.6 *ρ* = 0.94	RMSE = 2.88 SNR = 4.97 *ρ* = 0.95
11	RMSE = 4.28 SNR = 2.47 *ρ* = 0.77	RMSE = 3.54 SNR = 3.15 *ρ* = 0.86	RMSE = 3.8 SNR = 2.98 *ρ* = 0.88	RMSE = 3.2 SNR = 3.57 *ρ* = 0.91	RMSE = 2.61 SNR = 4.32 *ρ* = 0.93
13	RMSE = 3.38 SNR = 2.89 *ρ* = 0.81	RMSE = 2.64 SNR = 3.85 *ρ* = 0.89	RMSE = 2.2 SNR = 4.63 *ρ* = 0.94	RMSE = 1.98 SNR = 4.97 *ρ* = 0.95	RMSE = 1.94 SNR = 5.06 *ρ* = 0.95
14	RMSE = 5.34 SNR = 3.26 *ρ* = 0.83	RMSE = 5.56 SNR = 3 *ρ* = 0.85	RMSE = 7.36 SNR = 1.98 *ρ* = 0.82	RMSE = 5.08 SNR = 3.34 *ρ* = 0.89	RMSE = 4.36 SNR = 4 *ρ* = 0.92
17	RMSE = 2.22 SNR = 3.39 *ρ* = 0.86	RMSE = 2.05 SNR = 3.66 *ρ* = 0.89	RMSE = 4.3 SNR = 0.98 *ρ* = 0.75	RMSE = 2.69 SNR = 2.44 *ρ* = 0.83	RMSE = 1.97 SNR = 3.79 *ρ* = 0.91
18	RMSE = 5.6 SNR = 2.7 *ρ* = 0.76	RMSE = 4.73 SNR = 3.27 *ρ* = 0.86	RMSE = 5.86 SNR = 2.56 *ρ* = 0.87	RMSE = 5.01 SNR = 3.15 *ρ* = 0.88	RMSE = 3.52 SNR = 4.48 *ρ* = 0.93

Additionally, figure A11 presents scalp topographies of PSD, averaged across all semi-synthetic data participants for each method. These topographies are shown separately for the 1–8 Hz band (typically most affected by blink artifacts) and the 8–12 Hz, 12–25 Hz, and 25–40 Hz bands (typically less affected). The scalp maps reflect spatial distributions of PSD changes before and after artifact removal.
